# Impacts of Climate Change and Adaptation Strategies on Maize Yield in Kenya and Northwest China

**DOI:** 10.3390/plants15172709

**Published:** 2026-09-03

**Authors:** Jackson K. Koimbori, Shuai Wang, Jie Pan, Kuo Li, Liping Guo

**Affiliations:** 1 Key Lab for Agro-Environment, Ministry of Agriculture and Rural Affairs, Institute of Environment and Sustainable Development in Agriculture, Chinese Academy of Agricultural Sciences, Beijing 100081, China; jackson_kinyanjui@yahoo.co.uk (J.K.K.); panjie@caas.cn (J.P.); 2 College of Land and Environment, Shenyang Agricultural University, Shenyang 110866, China; shuaiwang666@syau.edu.cn (S.W.)

**Keywords:** adaptation strategies, CERES-Maize model, climate change, comparative regional analysis, maize yield

## Abstract

Climate change poses growing risks to maize-based food systems, yet comparative evidence across climatically similar regions on different continents remains limited. This study compares climate change impacts and adaptation responses in rainfed maize systems in Nakuru County, Kenya, and Northwest China—two geographically distant but climatically similar regions. The CERES-Maize model (DSSAT v4.7), the latest version available when this study was conducted, was calibrated and validated using observed phenology and grain yield data (2005–2009) from Agricultural Meteorological Experimental Stations. Independent evaluation confirmed excellent model performance, with a normalized root mean square error (*NRMSE*) of less than 10%. The model was driven by bias-corrected climate projections under RCP4.5 and RCP8.5 for the 2030s (2021–2040) and 2050s (2041–2060), relative to the 1986–2005 baseline. Changes in climate, maize phenology, and yield were quantified, and adaptation strategies were evaluated against the baseline. Warming and increased precipitation accelerated maize development, causing yield declines of 3–27% in Nakuru County and 5–22% in Northwest China, with larger losses under RCP8.5. Adaptation measures—including planting-date adjustments, supplemental irrigation, and late-maturing cultivars—increased yields by up to 39% in Kenya and 29% in Northwest China, with combined strategies outperforming individual measures. Coordinated, multi-strategy adaptation can offset a large share of projected yield declines, offering practical pathways to sustain maize productivity under future climate change.

## 1. Introduction

Global food security is increasingly threatened by climate change, with maize—a staple crop for billions—particularly vulnerable to rising temperatures and altered precipitation patterns. Although the impacts of climate change on maize production have been extensively studied at national and regional scales, comparative evidence across climatically similar but geographically distant regions remains scarce. This study examines Nakuru County, Kenya, and Northwest China together because, despite their geographical separation, both regions share critical features: heavy reliance on rainfed maize systems, high sensitivity to heat and water stress during flowering and grain filling, and comparable semi-arid to sub-humid climates. This parallel vulnerability makes them ideal candidates for a cross-continental comparison that tests the transferability of adaptation strategies across contrasting socio-economic and institutional contexts.

Agriculture in both regions is highly vulnerable to climate change. In Kenya, rainfed agriculture accounts for about 85% of production and contributes approximately 26% of national GDP, supporting 80% of rural livelihoods [1,2]. In Northwest China, maize is a cornerstone crop for livestock feed and regional food security, with production increasingly threatened by rising temperatures and changing precipitation patterns [3,4]. Both regions have experienced notable warming. Kenya’s average temperature has increased by +0.3 °C per decade between 1991 and 2021, contributing to an estimated 34% decline in agricultural productivity since 1961 [5,6], while Northwest China has warmed at 0.32 °C per decade from 1961 to 2021, with precipitation increasing by 9.32 mm per decade [7]. These regional and continental trends mirror broader global climate dynamics, as global surface temperatures have risen by 0.99 °C between 2001 and 2020 compared to 1850–1900 levels and are projected to increase further by 2.7–4.4 °C by 2080–2100 under different emission scenarios [8]. Such climate variability poses major risks to food security worldwide, particularly in climate-sensitive regions such as Africa and Asia [6,9,10].

Future climate projections in Kenya, based on downscaled Global Climate Models (GCM) data from Coupled Model Intercomparison Project Phase 5 (CMIP5) under RCP4.5, indicate increases in precipitation and temperature ranging from 7 to 48% and from 1 to 7 °C, respectively, for 2020–2100 relative to the 1986–2005 baseline [11,12]. In Northwest China, projections under RCP4.5 indicate average precipitation and temperature increases of 17.5% and 0.84 °C by the 2030s relative to the 2000s, with varying impacts on maize yields [13]. Similar studies in China and Pakistan show that climate warming can significantly alter crop growth stages and yield potentials, influenced by soil, cultivar, and management practices [14]. Northwest China is among the most vulnerable areas, with risks of low precipitation and high temperature, resembling many regions in Kenya. Kenya’s over-dependence on rainfed agriculture directly affects food security, a situation exacerbated by changing climate conditions [15,16]. Key climate factors affecting crop production in Africa include rainfall, temperature, climate variability, surface water runoff, and CO_2_ fertilization [17]. As a result of these projected changes [18], various future climate scenarios estimate mean annual maize yield increases under RCP4.5 and RCP8.5 of 2.51% and 2.3%, respectively, while a decrease of 2.07% is expected under RCP2.6 for 2021–2050 relative to 2012–2015 [19] (note: the 2012–2015 period refers to calibration/validation years in that study, whereas the present study uses 1986–2005 as a baseline). Adaptation is therefore essential to enhance agricultural resilience and reduce food insecurity, as agriculture is a major livelihood source for rural communities in Africa [20]. In Kenya, adaptation strategies include crop management (e.g., changing cultivars and planting dates) and conservation agriculture (e.g., irrigation, soil management, and water conservation) [11,21,22]. In Northwest China, adaptation under increasing temperatures will require efficient water resource use [23,24], along with delayed sowing to avoid heat stress during grain filling and heat-resistant hybrid cultivars [25].

While numerous studies have examined climate change impacts and adaptations on maize in single regions (e.g., Kenya or parts of China), comparative evidence across climatically similar but geographically distant regions remains limited. Pairing Nakuru County, Kenya, and Northwest China adds unique value by leveraging their analogous semi-arid to sub-humid conditions, rainfed maize dominance, and shared sensitivities to heat and water stress. This cross-continental comparison tests the transferability and generalizability of adaptation strategies—such as planting date shifts, supplemental irrigation, and late-maturing cultivars—beyond local contexts, providing broader insights for maize systems in vulnerable agroecological zones worldwide and addressing a key gap in the literature.

Given this context and the limited number of studies comparing climate change impacts and adaptation measures on maize production across climatically similar regions in Africa and China, the objectives of this study were to (i) quantify the impacts of future climate change on maize phenology and grain yield in Nakuru County, Kenya, and Northwest China under the RCP4.5 and RCP8.5 scenarios; (ii) evaluate the effectiveness of individual and combined adaptation strategies, including adjustments in planting dates, supplemental irrigation, and the use of improved cultivars; and (iii) compare maize responses between the two regions to assess the transferability and general applicability of adaptation strategies across climatically similar but geographically distant maize production systems. The findings are intended to provide scientific evidence to support climate-resilient maize production and inform adaptation planning and policy development in vulnerable agroecological regions.

## 2. Results

This section summarizes projected changes in key climate variables (temperature, precipitation, and solar radiation) under future climate scenarios, followed by the simulated impacts of these changes on maize phenology and grain yield. It then evaluates the effectiveness of selected adaptation measures, including planting-date adjustments, irrigation practices, cultivar replacement, and their combined effects, for both Nakuru County, Kenya, and Northwest China. All yield changes are reported as percentage differences relative to the 1986–2005 baseline. Each subsection includes an explicit comparison between Nakuru County, Kenya, and Northwest China. Unless otherwise stated, all simulated results are presented using the six calibrated genetic parameter sets (Par. 1–Par. 6) obtained through the GLUE calibration procedure. These parameter sets represent the six best-performing calibration solutions for the same maize cultivar and are presented to demonstrate the robustness of model projections while accounting for parameter uncertainty.

### 2.1. Climate Change Impacts and Adaptation Responses for Maize in Nakuru County, Kenya

#### 2.1.1. Projected Changes in Key Climate Variables (Temperature, Precipitation, and Solar Radiation) in the 2030s and 2050s

In Nakuru County, mean temperatures are projected to rise under both RCP4.5 and RCP8.5, with larger increases under RCP8.5, ranging approximately 0.6–1.5 °C in the 2030s and 1.1–3.4 °C in the 2050s relative to the 1986–2005 baseline. Annual precipitation is expected to increase under both scenarios, with slightly higher increases under RCP8.5. Solar radiation is projected to decrease by about 2–8% in the 2030s and 2–8% in the 2050s (Table S4; Figure S1). Compared to Northwest China (Table S6), Nakuru County experiences slightly lower absolute warming, smaller but more consistent precipitation increases, and a more uniform decrease in solar radiation.

#### 2.1.2. Effects of Future Climate Change on Maize Phenology (Flowering and Maturity)

In Nakuru County, maize phenology is projected to shorten under both RCP4.5 and RCP8.5, with earlier flowering and maturity across all cultivars (Figures S4 and S5; Table S4). Reductions in days to flowering range from about 1 day in the 2030s to 13 days in the 2050s, while time to maturity is reduced by 3 days in the 2030s and up to 34 days in the 2050s, with stronger effects under RCP8.5 due to higher temperatures and elevated CO_2_. The largest shifts occur at Njoro and Bahati, reflecting greater sensitivity to warming at these sites. Compared with Northwest China (Figures S15 and S16), Nakuru County shows a similar directional acceleration of phenology, but reductions in flowering and maturity duration are slightly smaller and more uniform across sites.

#### 2.1.3. Projected Impacts of Climate Change on Maize Grain Yield

**Figure 1:**
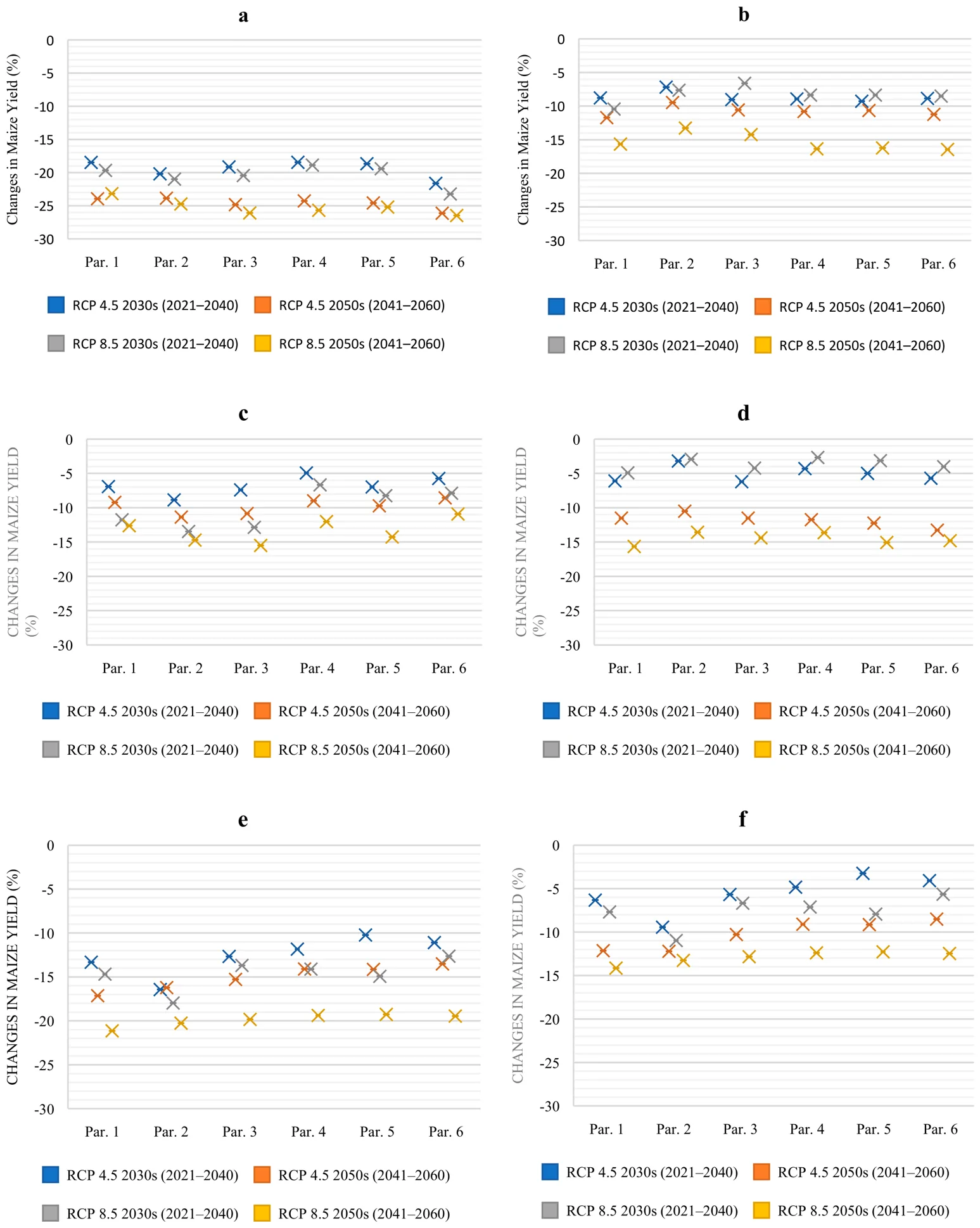
Projected changes in maize yield under the baseline climate (1986–2005) and future climate scenarios (2030s and 2050s) under RCP4.5 and RCP8.5 for the six study sites in Nakuru County, Kenya. Stations (**a**–**f**) represent Bahati, Gilgil, Molo, Naivasha, Njoro, and Subukia, respectively. Par. 1–Par. 6 denote the six highest-performing cultivar-specific genetic parameter sets obtained through the GLUE calibration procedure and used to represent calibration uncertainty in the simulations.

Without adaptation measures, mean maize grain yield in Nakuru County is projected to decline under both RCP4.5 and RCP8.5, with reductions ranging approximately 3–27% relative to the 1986–2005 baseline across the 2030s and 2050s (Figure 1). Yield losses are larger under RCP8.5 because of intensified heat stress and reduced solar radiation during critical growth stages, while elevated CO_2_ has a minimal effect on this C4 crop. In the CERES-Maize model, the response of C4 maize to elevated atmospheric CO_2_ is represented primarily through improved transpiration efficiency resulting from reduced stomatal conductance, rather than substantial increases in photosynthetic capacity or radiation-use efficiency. Consequently, the simulated CO_2_ fertilization effect on grain yield remains relatively small, consistent with the physiological characteristics of C4 crops. Compared with Northwest China (Figure 3), Nakuru County exhibits a similar directional yield decline relative to the baseline, but reductions are more uniform across sites, whereas Northwest China shows greater spatial variability and some mitigation from partial irrigation.

#### 2.1.4. Yield Responses to Adaptation Measures Under Future Climate Conditions

##### Effects of Planting Date Adjustments on Maize Yield

Shifting planting dates in Nakuru County shows clear directional effects on mean maize grain yield relative to the 1986–2005 baseline (Figures S6–S9). Advancing planting by 5–15 days generally reduces yield by up to 8–13% in the 2030s–2050s, whereas delaying planting increases yield by roughly 14–25%, depending on the scenario, period, and site. Delayed planting helps avoid thermal stress during flowering and grain filling, with larger gains under RCP8.5 because of greater temperature increases. Compared with Northwest China (Figures S17–S20), Nakuru County shows similar directional responses to planting-date shifts relative to the baseline, but the magnitude of yield increases from delayed planting is greater, reflecting the higher sensitivity of fully rainfed maize systems to temperature stress.

##### Effects of Irrigation on Maize Yield Under Different Planting Date Scenarios

Under supplemental irrigation in Nakuru County, mean maize grain yield relative to the 1986–2005 baseline shows minor reductions (roughly 0–10%) with early planting by 5–15 days, while delayed planting increases yield by about 13–22%, depending on scenario and period (Figures S10–S13). Yield gains are generally higher under RCP8.5, and combining irrigation with delayed planting maximizes benefits. Compared with Northwest China (Figures S21–S24), Nakuru County exhibits larger yield gains from delayed planting combined with irrigation relative to the baseline, reflecting the greater sensitivity of rainfed systems to temperature and water stress, whereas Northwest China shows more moderate benefits because of existing irrigation coverage.

##### Effects of Cultivar Replacement on Maize Yield

Replacing medium-maturing cultivars (H519, H513) with the late-maturing H6218 in Nakuru County increases mean maize grain yield by roughly 8–22% relative to the 1986–2005 baseline, depending on scenario (RCP4.5 or RCP8.5) and site (e.g., Njoro or Gilgil) (Figure S14). Gains are larger under RCP4.5, as the longer growing period allows better exploitation of moderate warming. Compared to Northwest China (Figure S25), Nakuru County shows similar directional gains from late-maturing cultivars relative to the baseline, but absolute percentage increases are slightly higher because rainfed maize is more responsive to warming.

##### Combined Effects of Multiple Adaptation Measures on Maize Yield

**Figure 2:**
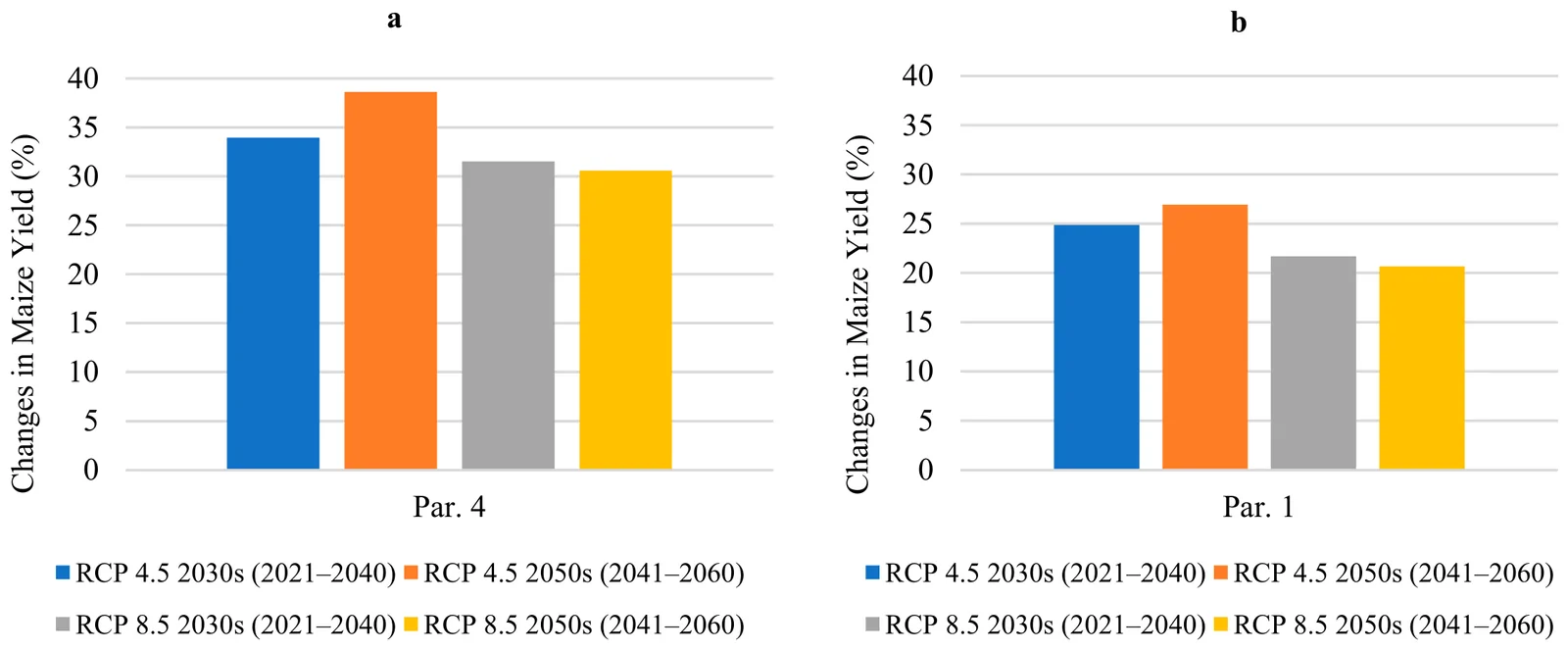
Projected maize yield changes from combined adaptation strategies (delayed planting, supplemental irrigation, and late-maturing cultivars) under future climate scenarios (2030s and 2050s) for the six study sites in Nakuru County, Kenya. Stations (**a**,**b**) represents Njoro and Gilgil respectively. Par. 1–Par. 6 denote the six highest-performing cultivar-specific genetic parameter sets obtained through the GLUE calibration procedure and used to represent calibration uncertainty in the simulations.

Combining delayed planting by 5–15 days, appropriate supplemental irrigation, and replacement with the late-maturing cultivar H6218 in Nakuru County is projected to increase mean maize grain yield by roughly 25–39% relative to the 1986–2005 baseline, depending on site and scenario (Figure 2). The largest gains occur at Njoro, reflecting high sensitivity to temperature and rainfed conditions. This demonstrates that integrated adaptation measures can fully offset or exceed projected climate-induced declines relative to the baseline. Compared to Northwest China (Figure 4), Nakuru County shows similar positive responses to combined adaptations relative to the baseline, but percentage gains are slightly higher and more uniform, while Northwest China gains are more site-dependent due to partial irrigation coverage and regional climate differences.

### 2.2. Climate Change Impacts and Adaptation Responses for Maize in Northwest China

#### 2.2.1. Projected Changes in Key Climate Variables (Temperature, Precipitation, and Solar Radiation) in the 2030s and 2050s

In Northwest China, mean temperature is projected to rise by roughly 0.5–3.3 °C relative to the 1986–2005 baseline, precipitation is expected to change by about −2 to +54% depending on scenario and site, and solar radiation is projected to decline slightly by 0.4–3.3% (Table S6). Changes are generally larger under RCP8.5 and in the 2050s. Compared to Nakuru County (Table S4), Northwest China exhibits similar directional trends for warming and precipitation increases, but absolute temperature rises are slightly higher and precipitation changes are more variable across sites.

#### 2.2.2. Effects of Future Climate Change on Maize Phenology (Flowering and Maturity)

In Northwest China, maize phenology accelerates under climate change, with days to flowering decreasing by ~1–17 days and time-to-maturity shortening by ~3–38 days relative to the baseline, depending on site, cultivar, and scenario (Figures S15 and S16). Reductions are larger under RCP8.5 due to higher temperatures and CO_2_ levels, with greatest declines at Hami, Jingyuan, and Yulin. Compared to Nakuru County (Figures S4 and S5), phenological shortening is similar in direction, but the magnitude is slightly larger in Northwest China and affects more sites.

#### 2.2.3. Projected Impacts of Climate Change on Maize Grain Yield

**Figure 3:**
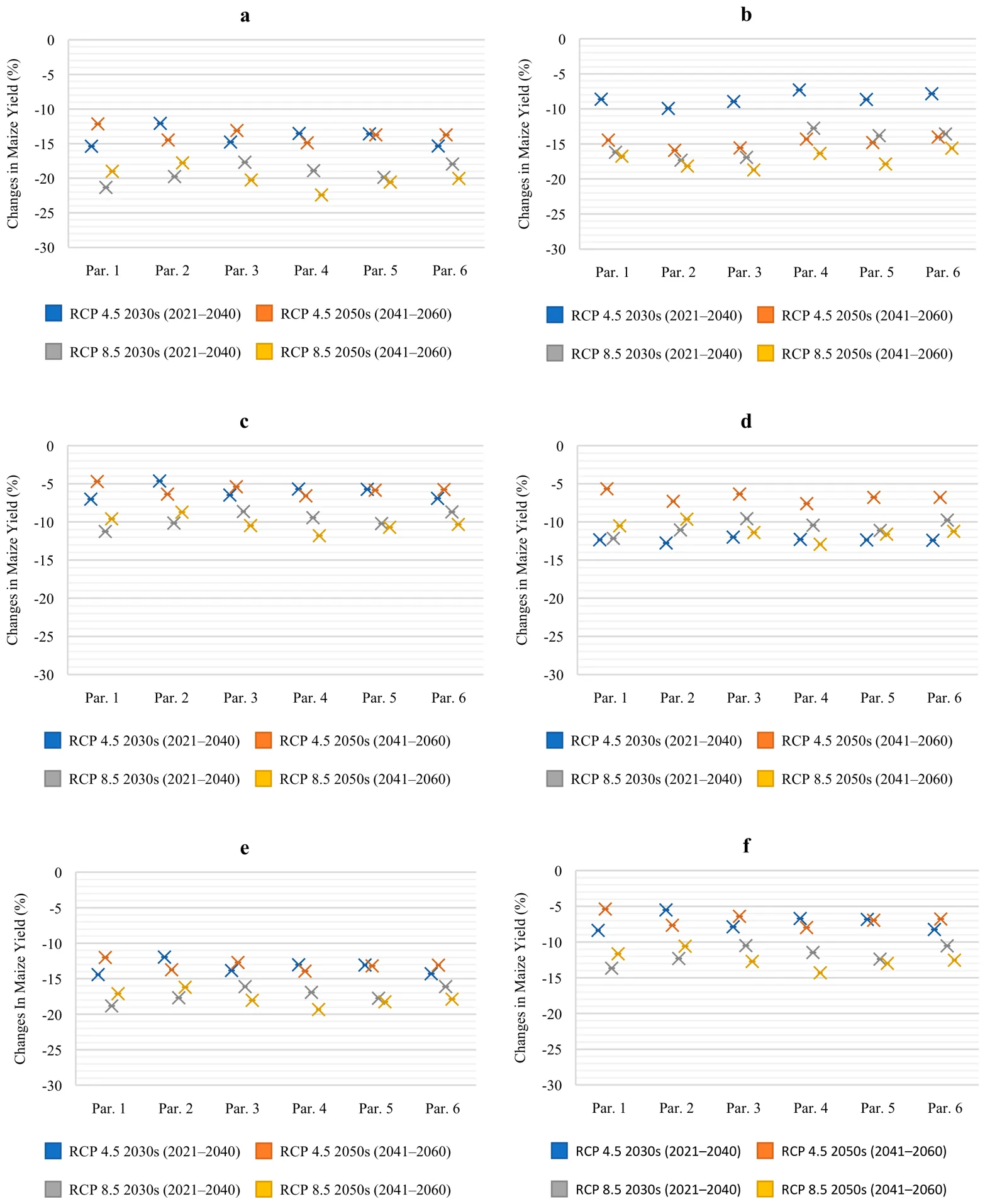
Projected maize grain yield under the baseline climate (1986–2005) and future climate scenarios (2030s and 2050s) under RCP4.5 and RCP8.5 for the six study sites in Nakuru County, Kenya. Stations (**a**–**f**) represent Bahati, Gilgil, Molo, Naivasha, Njoro, and Subukia, respectively. Par. 1–Par. 6 denote the six highest-performing cultivar-specific genetic parameter sets obtained through the GLUE calibration procedure and used to represent calibration uncertainty in the simulations.

Without adaptation, average maize grain yield in Northwest China declines under climate change, with reductions ranging approximately 4–22% relative to the 1986–2005 baseline across sites and scenarios (RCP4.5 and RCP8.5) (Figure 3; Table S6). The largest declines occur at Hami (~22%) and Jingyuan (~19%), primarily due to increased temperature and reduced solar radiation. Elevated CO_2_ has little effect, consistent with maize as a C4 crop. Compared to Nakuru County (Figure 1), Northwest China shows slightly lower but more variable yield reductions relative to the baseline, whereas Kenya’s fully rainfed maize exhibits more uniform losses.

#### 2.2.4. Yield Responses to Adaptation Measures Under Future Climate Conditions

##### Effects of Planting Date Adjustments on Maize Yield

In Northwest China, delaying planting by 5–15 days generally increases mean maize grain yield relative to the 1986–2005 baseline, with gains up to ~18% under RCP4.5 in the 2050s, while advancing planting can reduce yield by up to ~25% under RCP8.5 (Figures S17–S20). Delayed planting helps avoid thermal stress during key stages. Compared to Nakuru County (Figures S6–S9), yield gains from delayed planting relative to the baseline are slightly smaller in Northwest China, reflecting lower sensitivity to seasonal temperature changes.

##### Effects of Irrigation on Maize Yield Under Different Planting Date Scenarios

Under irrigation in Northwest China, early planting by 5–15 days generally reduces mean maize grain yield relative to the 1986–2005 baseline, while delayed planting increases yield by roughly 0–17%, with early planting decreases of 0–16.5% depending on scenario and period (Figures S21–S24). Delayed planting under irrigation helps avoid thermal stress. Compared to Nakuru County (Figures S10–S13), Northwest China shows similar directional responses relative to the baseline, but the magnitude of gains with irrigation is smaller, reflecting higher vulnerability of rainfed maize in Kenya.

##### Effects of Cultivar Replacement on Maize Yield

Replacing medium-maturing cultivars with the late-maturing cultivar Xinyu No. 18 consistently increases mean maize grain yield in Northwest China by 11–19% under RCP4.5 and 8–12% under RCP8.5 relative to the 1986–2005 baseline across the 2030s and 2050s, depending on site and parameters (Figure S25). The longer growing period allows better use of higher temperatures. Compared to Nakuru County (Figure S14), absolute yield gains from cultivar replacement relative to the baseline are slightly lower in Northwest China, reflecting greater sensitivity of rainfed maize in Kenya to warming.

##### Combined Effects of Multiple Adaptation Measures on Maize Yield

**Figure 4:**
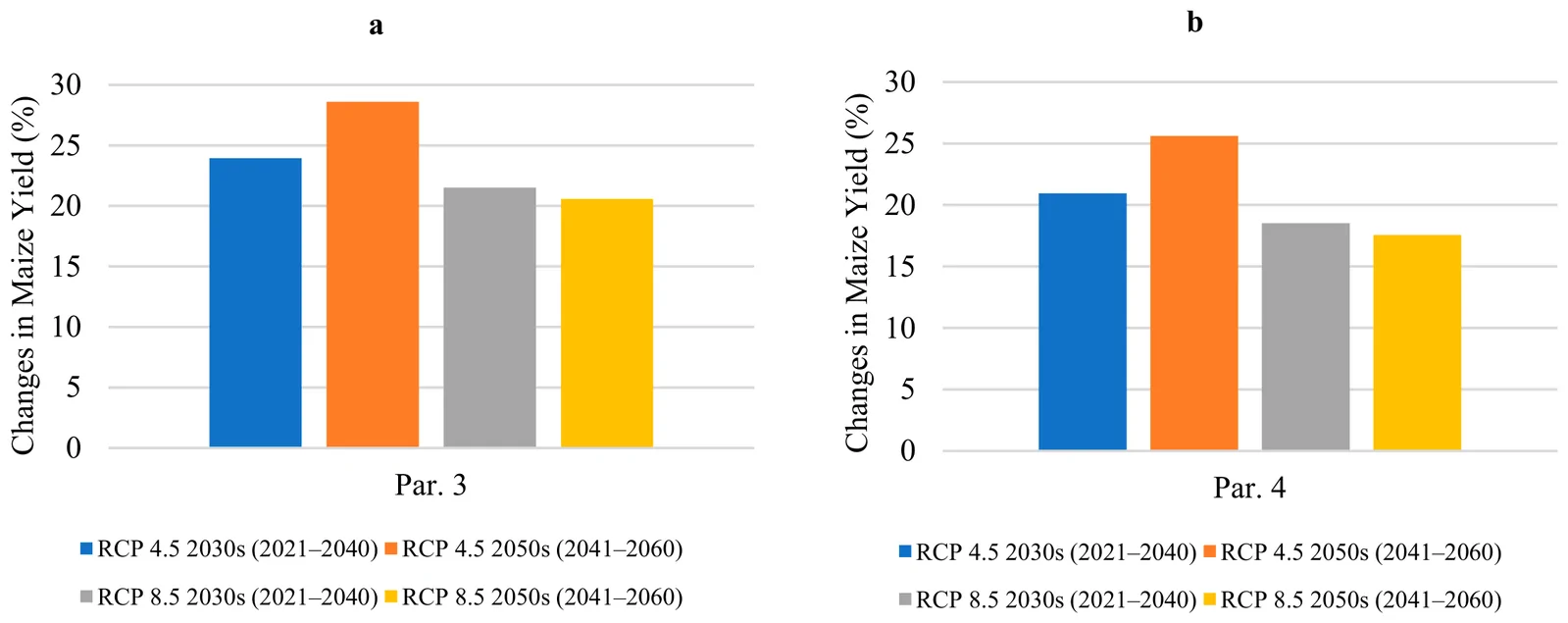
Projected changes in maize yield under different climate change scenarios and adaptation strategies for the 2030s (2021–2040) and 2050s (2041–2060) under RCP4.5 and RCP8.5 for the six study sites in Nakuru County, Kenya. Stations (**a**,**b**) represents Hami and Jingyuan respectively. Bars represent the mean simulated responses across the six calibrated genetic parameter sets (Par. 1–Par. 6) obtained through the GLUE calibration procedure.

Combining delayed planting by 5–15 days, appropriate irrigation, and the late-maturing cultivar Xinyu No. 18 produces the largest increases in mean maize grain yield in Northwest China relative to the 1986–2005 baseline. At Hami, gains range 24–29% under RCP4.5 and 21–22% under RCP8.5 for the 2030s and 2050s; at Jingyuan, gains are 21–26% under RCP4.5 and 18–18% under RCP8.5 (Figure 4). These results show integrated adaptation can leverage warming for productivity gains relative to the baseline. Compared to Nakuru County (Figure 2), combined measures lead to slightly smaller maximum yield gains relative to the baseline in Northwest China, reflecting compounded benefits in Kenya’s temperature-sensitive, rainfed maize systems.

### 2.3. Relative Contributions of Climate Variables to Yield Changes

To isolate the relative contributions of temperature, precipitation, and solar radiation to projected yield changes, we conducted additional sensitivity simulations for Nakuru County and Northwest China under RCP8.5 (2050s). Each climate variable was individually perturbed while holding others at baseline levels (1986–2005). Results indicated that temperature increases were the dominant driver of yield decline, accounting for approximately 65–78% of total yield reduction in Nakuru County and 58–72% in Northwest China. Reduced solar radiation contributed 18–28% (Kenya) and 22–35% (Northwest China) of yield losses, primarily through shortened grain-filling periods and reduced photosynthesis. Precipitation changes had mixed effects: increased rainfall partially offset thermal stress in some sites (contributing +5 to +12% yield recovery), but variability and intense events caused waterlogging losses in others (−3 to −8%). Net precipitation effects were thus regionally heterogeneous, ranging from −5% to +8% of total yield change in Kenya and −10% to +15% in Northwest China. These findings confirm that warming is the primary constraint on future maize productivity in both regions, with radiation declines exacerbating losses and precipitation changes playing a secondary, site-dependent role (Table S4; Table S6).

## 3. Discussion

Maize is a staple food crop in both Kenya and Northwest China, playing a critical role in food security and regional economies. In Kenya, maize is highly vulnerable to climate variability [26]. Our results show that projected increases in temperature (0.58–3.35 °C) and precipitation (3.01–11.76%), along with declining solar radiation (−1.15 to −8.05%) in Nakuru County during the 2030s and 2050s under RCP4.5 and RCP8.5, would reduce maize yield by 2.7–26.5% and shorten growth duration by 1–34 days. Sensitivity analysis (Section 2.3) revealed that temperature increases were the dominant driver of these declines, accounting for approximately 65–78% of total yield loss, followed by reduced solar radiation (~18–28%), while precipitation changes had net neutral to slightly positive effects depending on site. In the Western and Central Highlands, yield reductions are projected to average 10.8% (2050s) and 23.7% (2070s), with site-specific extremes ranging from 3.2% (Naivasha, parameter 2) to 26.1% (Subukia, parameter 6) [27], consistent with previous projections using five CMIP5 RCP8.5 climate models [27].

Similarly, in Northwest China, maize yield reductions were primarily influenced by increasing temperatures between 1979 and 2016 [28]. Our study projects temperature increases of 0.48–3.26 °C, precipitation increases of 2.34–54.31%, and solar radiation declines of −0.40 to −3.31% during the 2030s and 2050s under RCP4.5 and RCP8.5. These changes would shorten maize growth duration and reduce yields, with the lowest and highest reductions recorded in Hetian and Hami (4.6% under parameter 2 and 22.4% under parameter 4). Decomposition analysis attributed 58–72% of yield decline to elevated temperatures, 22–35% to reduced solar radiation, and the remainder to precipitation variability, with drought stress at arid sites (Hami, Jingyuan) offsetting gains from increased rainfall at wetter sites (Hetian, Changji). These results are consistent with previous studies that projected temperature increases of approximately 6 °C, precipitation increases of more than 50 mm, and declining daily solar radiation using the RegCM4.6 regional climate model, resulting in reduced maize yields [29,30]. Additional research also supports the observed trends in temperature increases during the maize growth period and associated yield declines [28,31,32].

Comparing Nakuru County and Northwest China reveals both similarities and differences that have implications for generalizing adaptation strategies. Both regions are climatically sensitive, with maize yields strongly affected by temperature and precipitation variability. However, the relative contributions differ: Kenya shows higher temperature dominance (65–78% vs. 58–72%) due to fully rainfed systems where thermal stress is unmitigated by water management, while Northwest China exhibits slightly higher radiation contributions (22–35% vs. 18–28%) due to stronger baseline solar dependency in arid environments. Nakuru County experiences higher rainfall variability and a wider range of solar radiation changes, while Northwest China faces more extreme aridity, lower mean annual rainfall, and larger temperature fluctuations. These differences explain why combined adaptation strategies (delayed planting by 5–15 days, supplemental irrigation, and late-maturing cultivars) produce higher relative yield gains in Nakuru County (up to ~39%, as shown in Figure 2) than in Northwest China (up to ~29%, as shown in Figure 4). In Nakuru County’s fully rainfed systems, greater baseline sensitivity to temperature-driven stress and rainfall variability during reproductive stages makes delayed planting more effective at avoiding peak heat, and supplemental irrigation more transformative in addressing acute water deficits (Table S4). In Northwest China, higher absolute warming and site-specific precipitation variability (up to +54%) allow late-maturing cultivars to exploit extended thermal resources, but gains are moderated by existing partial irrigation, which reduces marginal benefits from additional water applications (Table S6) [3,21,23,24,33]. Adaptation transferability is therefore high for planting date adjustments and cultivar changes (directionally consistent), but irrigation is more critical in Northwest China’s arid context for baseline stability and more impactful in Kenya’s rainfed systems for offsetting climate-induced deficits.

Adjustment of planting dates is already practiced by many farmers in response to seasonal rainfall variability. Our findings suggest that further optimization of sowing dates under future climate conditions could provide additional yield benefits, although the optimal planting window will remain location-specific and dependent on rainfall patterns, temperature, and cropping calendars. Adaptation measures can substantially mitigate the negative impacts of climate change on maize yields in both regions. In Kenya, delaying planting dates by 5–15 days was the most effective single adaptation, increasing maize yields by 14.3–21.4%, while combining adaptation measures, including hybrid cultivars and improved soil fertility, resulted in yield gains of 20.7–38.6% under RCP4.5 and RCP8.5 during the 2030s and 2050s [1,11,21]. The effectiveness of delayed planting directly reflects the dominant role of temperature stress (Section 2.3), by shifting reproductive stages to cooler periods, this strategy addresses the primary yield-limiting factor. In Northwest China, irrigation was the most effective adaptation measure, improving yields by 15.9–19.8%, while combinations with other adaptation measures increased yields by 17.6–28.6% under the same scenarios [3,9,34,35]. However, expansion of irrigation should be carefully balanced against sustainable water management objectives, since increased irrigation may increase energy requirements and associated greenhouse gas emissions where fossil-fuel-powered pumping systems are used. Improving irrigation efficiency through water-saving technologies can help maximize climate adaptation benefits while minimizing environmental impacts. These findings are consistent with prior research suggesting that integrated crop management, cultivar changes, and irrigation practices are effective strategies to enhance maize yield under climate stress [21,22,34].

From a practical perspective, implementation of these adaptation measures will depend on local farming systems and resource availability. In Nakuru County, supplemental irrigation remains limited because most maize production is rainfed and constrained by water availability and investment costs, whereas parts of Northwest China already benefit from existing irrigation infrastructure. Consequently, delayed planting and improved cultivars are likely to represent the most immediately scalable adaptation options, while irrigation expansion should be implemented only where water resources can be sustainably managed.

Despite these positive outcomes, limitations remain and affect confidence in specific findings. The study did not consider economic factors, farmer adoption feasibility, or local market constraints, which could reduce the practical effectiveness of adaptation strategies below simulated biophysical potentials. Projections carry uncertainty due to potential biases in climate models (e.g., precipitation variability in HadGEM2-ES outputs) and limitations in representing all soil processes in the CERES-Maize model, potentially overestimating upper-end gains (~39% in Kenya, ~29% in China), especially under RCP8.5 or with unmodeled stresses like pests or extremes [11,12,13]. Additionally, our reliance on a single GCM (HadGEM2-ES) introduces uncertainty, as multi-model ensembles typically show wider spreads in precipitation projections and temperature extremes than any single model; future studies should incorporate CMIP6 ensembles to better quantify projection uncertainty [13]. Furthermore, the relative contribution analysis (Section 2.3) assumes additive effects of climate variables; interactions between temperature, water stress, and radiation may produce non-linear responses not captured by single-factor perturbations. Recognizing these limitations is essential when interpreting results and designing region-specific interventions. Genetic improvement also represents an important complementary adaptation pathway. Development and wider adoption of heat-tolerant, drought-tolerant, and longer-duration maize cultivars can enhance resilience under future climate conditions by maintaining grain filling and reducing heat stress during reproductive stages. Integrating improved cultivars with optimized planting dates and efficient irrigation is likely to provide greater resilience than any single adaptation measure alone.

The findings have important implications for practice and future research. Given that temperature increases dominate yield declines in both regions (~60–75% of total loss), adaptation policies should prioritize heat avoidance strategies (delayed planting, heat-tolerant cultivars) alongside water management. However, realizing these biophysical potentials requires concurrent investments in: (i) water infrastructure and governance to ensure sustainable irrigation supply; (ii) seed systems and subsidy mechanisms to improve cultivar access; and (iii) farmer extension and risk management tools to overcome adoption barriers. In Nakuru County, Kenya, agricultural extension programs should immediately revise planting calendars to delay sowing by 10–15 days, paired with seed system subsidies for late-maturing cultivar H6218 and small-scale water harvesting infrastructure to enable supplemental irrigation. In Northwest China, policies should prioritize irrigation efficiency upgrades (drip/micro-sprinkler), mandated transition to heat-tolerant cultivars Xinyu No. 18 at water-stressed sites (Hami, Jingyuan), and climate-informed water allocation planning. Future studies should integrate biophysical modeling with socio-economic assessments of farmer adoption, irrigation feasibility, and breeding strategies to develop adaptation pathways that are both agronomically effective and practically implementable under future climate conditions.

## 4. Materials and Methods

### 4.1. Study Areas in Kenya and China

#### 4.1.1. Nakuru County in Kenya

The study area of Nakuru County (Figure S1), which is located in Southwest Kenya, consists of 11 sub-counties, of which 6 were selected for study in this paper, including Bahati, Gilgil, Molo, Naivasha, Njoro, and Subukia, since the other 5 sub-counties are non-agricultural dominated. The greater Nakuru County is in the Rift Valley Region and occupies an area of 7242.3 km^2^ with a population of 2,162,202 [36]. The county lies approximately between latitudes 0°18′ S and 1°10′ S and longitudes 35°28′ E and 36°00′ E, at an average altitude of about 1912 m above sea level. The climate of Nakuru County covers four zones that are strongly influenced by altitude and physical features, with mean annual rainfall ranging between 500 mm and 2700 mm and temperatures ranging between 10 °C and 20 °C during the cold months (July and August) and during the hot months (January to March), respectively [21]. Maize production in Nakuru County accounts for about 61% of the total crop production, making Nakuru County one of the food baskets of Kenya [37]. The county’s soil type presents a complex distribution of three main classifications that have been influenced by climatic conditions, volcanic activities, and the underlying rock type. The three major soil types in the county are Latosolic, Planosolic, and Alluvial [38].

#### 4.1.2. Northwest China

The study area, Northwest China (Figure S2), consists of 5 provinces, namely Shaanxi, Xinjiang, Gansu, Qinghai, and Ningxia, covering a total area of approximately 3,100,000 km^2^ and with a population of around 103.5 million [39]. NWC spans longitudes from about 73° E to 111° E and latitudes from about 30° N to 49° N, and boasts various mountains such as the Qilianshan Mountains, Helanshan Mountains, Wushaoling Mountains, Tianshan Mountains, Altai Mountains, and Kunlun Mountains that could have an impact on the regional climate [40]. NWC has various basins located in the rain shadow of these mountains, such as the Junggar Basin, Tarim Basin, and Qaidam Basin [40,41].

The climate in NWC is a typical temperate continental climate, with an annual mean temperature of 8 °C, an annual mean rainfall of less than 200 mm, and an annual mean evaporation of 1291 mm [40,41,42]. In Northwest China, maize is one of the main crops grown, contributing to more than 50% of the region’s total grain yields and covering more than 30% of the total grain planting area, thereby playing a pivotal role in the regional agricultural economy [43]. A vast area in NWC is characterized by continental arid conditions, partly attributed to the presence of deserts such as the Taklimakan Desert, Gurbantunggut Desert, Qaidam Desert, and Tengger Desert [39]. The soil type of the region is Siltigi-Orthic Anthrosols, which developed over time from Calci-Orthic Aridisols due to long-term application of fertilizer, cultivation, and mineral-rich sediment water [44].

### 4.2. CERES-Maize Model

The most recent version of CERES-Maize (DSSAT v4.7) crop model was used to simulate spring maize growth, development, and yield in both Kenya and China. The model integrates weather, soil, crop management, and genotype/phenotype data and offers enhanced functionality compared to earlier versions [45]. Inputs include crop management practices (cultivar, planting date, density, row spacing, fertilizer type/rate, irrigation method/amount, and tillage) [46], daily weather data (minimum/maximum temperature, precipitation, humidity, solar radiation) [46], and soil data per layer (type, texture, pH, moisture, organic carbon, nitrogen, bulk density, cation exchange capacity, drainage) [47]. Weather data were obtained from AMESs via the China Meteorological Data Network (http://data.cma.cn/; accessed on 15 March 2020) and the Kenya Meteorological Station (https://meteo.go.ke/; accessed on 15 March 2020), with daily solar radiation calculated from sunshine hours using the Angstrom–Prescott equation [45]. Future climate data were generated using the HadGEM2-ES GCM at 0.5° × 0.5° resolution and bias-corrected before incorporation. Soil data were obtained from AMESs, the Chinese Soil Scientific Database, and the Kenya Soil Scientific Database. Crop management data from 2005 to 2009 included cultivar, planting date, density, spacing, fertilizer/irrigation practices, tillage, and harvest dates [46,47].

#### 4.2.1. Site Selection Criteria for Modeling

Sites for calibration and validation were selected based on three main criteria: (i) maize cultivars must have been cultivated for at least three consecutive years without significant stress from pests, diseases, or severe climatic events; (ii) field management practices (sowing dates, row spacing, cultivar changes, fertilizer/irrigation rates, pest and disease treatments) were well-documented and kept consistent across all scenarios except for adaptation treatments; and (iii) study sites were representative of sub-areas and located near major AMESs to ensure access to accurate weather data. The selected stations are listed in Tables S1 and S2.

The cultivars used in the Chinese study sites were developed and supplied by local provincial and city agricultural research institutes under the National Key R&D Program of China and the Agricultural Science and Technology Innovation Program of the Chinese Academy of Agricultural Sciences (CAAS). These included cultivars such as 4zao6, Haiyu 6, Jidan 159, Limin15, Lianyu 6, Dongdan 60 (Northeast China), and SC704, Xinyu No. 18, Shen Yu 2002, 296, Shendan No. 16, and Shendan No. 10 (Northwest China). In Kenya, the cultivars H6218, H513, H6210, and H520 were developed and provided by the Kenya Agricultural and Livestock Research Organization (KALRO), the national agricultural research institute responsible for maize genetic improvement in Kenya. All cultivars were selected based on their regional adaptation and documented performance in multi-environment trials.

#### 4.2.2. Parameterization and Genetic Coefficients

Genetic coefficients in the CERES crop model are defined as a set of parameters that describe the interaction between crop genotype and environmental conditions [1]. In the CERES-Maize (DSSAT v4.7) model, six genetic coefficients (P1, P2, P5, G2, G3, and PHINT) are used to characterize a maize cultivar (Table S3), with prior ranges for GLUE calibration defined as follows: P1 (100–400 °C·day), P2 (0.0–4.0 day), P5 (400–990 °C·day), G2 (200–990 kernels per plant), G3 (3.0–20.0 mg·day^−1^), and PHINT (30–70 °C·day·leaf^−1^). These coefficients govern crop phenology, growth, and development processes [46,47]. DSSAT incorporates a coefficient estimator module known as the Generalized Likelihood Uncertainty Estimation (GLUE), which was used to estimate genotype-specific coefficients for each maize cultivar in this study [48,49]. For each cultivar, a unique set of six genetic coefficients was derived by calibrating simulated outputs against observed phenological duration (days to flowering and maturity) and grain yield data from 2005 to 2009. Among the six genetic coefficients, four parameters (P1, P2, P5, and PHINT) primarily control the timing of phenological stages, while the remaining two parameters (G2 and G3) define potential yield under optimal growth conditions.

The six retained parameter sets (Par. 1–Par. 6) correspond to the six best-performing combinations of genetic coefficients identified during GLUE calibration. These parameter sets are used consistently throughout the manuscript to quantify uncertainty associated with genotype parameter estimation. They should therefore be interpreted as alternative calibrated representations of the same maize cultivar rather than different cultivars.

#### 4.2.3. Model Calibration

Calibration was conducted by applying six independent sets of cultivar-specific genetic coefficients generated using the Generalized Likelihood Uncertainty Estimation (GLUE) procedure for each maize cultivar at every study site. The six retained parameter sets (Par. 1–Par. 6) for each cultivar are presented in Supplementary Table S5. These represent the six highest-performing GLUE calibration solutions identified for each cultivar and were retained to account for parameter uncertainty in subsequent simulations. Rather than selecting a single optimum parameter combination, the six highest-performing parameter sets (Par. 1–Par. 6) were retained because they all produced satisfactory agreement with observed flowering, maturity, and grain yield while representing parameter uncertainty within the calibration process. Each parameter set therefore represents an equally acceptable calibration solution rather than a different cultivar or management treatment. All subsequent simulations were performed using these six calibrated parameters sets, and the results are presented to demonstrate the robustness of model projections to calibration uncertainty. Simulated flowering, maturity, and grain yield were compared with observed data. In Nakuru County, *NRMSE* ranged from 0.8 to 9.0%, with *PD* ranging from −10.0 to 9.6%, while in Northwest China, *NRMSE* ranged from 0.7 to 5.9%, with *PD* ranging from −3.4 to 2.0%, indicating excellent model performance.

#### 4.2.4. Evaluation

Independent evaluation was conducted using observed data not applied during the calibration process. Simulated phenology and grain yield were compared against independent datasets to assess model robustness under varying soil and management conditions in both Kenya and Northwest China. Model performance was assessed using two statistical indicators: (i) normalized root mean square error (*NRMSE*), which quantifies the relative magnitude of simulation error; and (ii) predicted deviation (*PD*), which indicates the direction of bias (over- or under-estimation). Model performance was classified as excellent when *NRMSE* < 10%, good when 10–20%, fair when 20–30%, and poor when >30%. A negative *PD* value indicates under-prediction, whereas a positive value indicates over-prediction. The evaluation metrics were calculated as follows:

$$NRMSE=\frac{{\left[\;\sum_{i=1}^{n}\frac{{\left(S_{i}-O_{i}\right)}^{2}}{n}\;\right]}^{0.5}}{\bar{o}}$$

$${PD}_{i}=\left(\frac{S_{i}-O_{i\;}}{O_{i}}\right)*100$$

where $s_{i}$ and $o_{i}$ are simulated and observed variables, respectively; $\bar{o}$ is the mean value of the observed data; n is the number of comparisons; and *i* denotes each comparison.

### 4.3. Baseline and Scenario Simulations

#### 4.3.1. Baseline Simulation

The baseline simulation (1986–2005) was defined as the reference for all comparisons, including future climate impacts and adaptation treatments. Inputs included historical daily weather data (maximum and minimum temperature, precipitation, humidity, and solar radiation), site-specific soil data (texture, pH, organic carbon, nitrogen, bulk density), and crop management data (cultivar, planting date/density/spacing, fertilizer type/rate, irrigation method/amount, tillage, and harvesting date). Assumptions included fixed management practices (except for adaptation treatments), no major biotic or abiotic stresses, and rainfed conditions unless specified. The baseline simulation period was defined as 1986–2005, following the IPCC AR5 standard reference for CMIP5-based climate impact assessments, ensuring protocol consistency with the HadGEM2-ES projections used in this study and methodological alignment with international assessment standards.

#### 4.3.2. Climate Scenario Selection and Future Scenario Simulations

Future climate scenarios included RCP4.5 (medium emissions) and RCP8.5 (high emissions) based on projected 2100 radiative forcing and greenhouse gas pathways [13,50,51]. Daily weather projections for each scenario were generated using HadGEM2-ES at 0.5° × 0.5° resolution and bias-corrected before incorporation. Future simulations incorporated dynamic atmospheric CO_2_ concentrations corresponding to each Representative Concentration Pathway (RCP4.5 and RCP8.5) using the HadGEM2-ES climate projections. Consequently, atmospheric CO_2_ levels varied according to the respective emissions pathway and simulation period rather than remaining fixed at baseline conditions. These data were downloaded from https://pcmdi.llnl.gov/?cmip5 and https://esgf-node.llnl.gov/projects/esgf-llnl/ (accessed on 15 March 2020).

#### 4.3.3. Sensitivity Analysis

A sensitivity analysis was conducted to evaluate the relative contribution of individual climatic variables to projected maize yield changes. Single-factor perturbation simulations were performed by varying one climatic variable (temperature, precipitation, or solar radiation) at a time while keeping all other climatic variables, soil characteristics, crop management practices, and cultivar genetic coefficients unchanged from the baseline simulation. The CERES-Maize model was then run under each perturbation for the RCP4.5 and RCP8.5 scenarios during the 2030s (2021–2040) and 2050s (2041–2060). Simulated grain yields from each perturbation were compared with the baseline simulation to quantify the relative influence of each climatic variable on maize productivity.

#### 4.3.4. Adaptation Simulations

Adaptation simulations tested individual and combined measures relative to the baseline (1986–2005 conditions), keeping all other inputs fixed unless explicitly modified. Fixed inputs included soil properties (texture, pH, organic carbon, nitrogen levels, bulk density, cation exchange capacity, drainage, etc.), fertilizer type, rate, timing, and method, planting density, row spacing, planting depth and configuration, tillage practices, harvesting assumptions, initial soil conditions (water and nitrogen), crop type, and absence of pest/disease stresses.

##### Planting Date Adjustments

Planting dates were advanced or delayed by 5–15 days to reduce thermal stress during flowering and grain filling (Figures S6–S9 and S17–S20). Two directional shifts were tested relative to baseline sowing dates: (i) advanced planting (5, 10, and 15 days earlier) to exploit cooler early-season conditions, and (ii) delayed planting (5, 10, and 15 days later) to avoid peak heat during reproductive stages. Baseline planting dates were derived from observed management records (2005–2009): mid-March to early April for Nakuru County, Kenya, and mid-April to early May for Northwest China. Adjusted dates were constrained to remain within viable sowing windows (soil temperature > 10 °C at 10 cm depth, no forecasted frost events). All other management parameters (density, spacing, fertilizer) were held constant across planting date treatments to isolate phenological effects.

##### Supplemental Irrigation

Supplemental irrigation was applied under different planting date scenarios to mitigate water stress, with amounts, timing, and methods optimized for each site, scenario, and future period under RCP4.5 and RCP8.5 (Figures S10–S13 and S21–S24). Irrigation events were triggered based on soil moisture thresholds (50% of plant-available water capacity in the root zone) and crop phenological stages to maximize water use efficiency. In Nakuru County, Kenya, irrigation amounts ranged from 50 to 100 mm per application, delivered at flowering and grain-filling stages, reflecting infrastructure constraints in rainfed smallholder systems. In Northwest China, irrigation amounts ranged from 80 to 150 mm per application, with 2–3 events timed to avoid overlap with natural precipitation peaks. The DSSAT automatic irrigation routine was configured with a 5-day application window, and seasonal totals did not exceed 300 mm (Kenya) or 450 mm (Northwest China) to represent realistic water availability. Irrigation water was assumed to be 100% efficient (no conveyance losses) to isolate biophysical yield responses.

##### Cultivar Replacement

Medium-maturing cultivars such as H519 and H513 in Nakuru County, Kenya, and SC704 at Hami and Shendan No. 16 at Jingyuan in Northwest China were replaced with late-maturing cultivars H6218 in Kenya and Xinyu No. 18 in Northwest China by updating genetic coefficients to extend the growth period and better exploit increased temperatures (Table S3; Figures S14 and S25). Genetic coefficients were derived from GLUE calibration against observed phenology and yield data (2005–2009) from variety trials. In Kenya, H6218 parameters were: P5 = 780 °C·day, G2 = 850 kernels plant^−1^, G3 = 12.5 mg day^−1^ (vs. baseline H519/H513: P5 = 620–680 °C·day). In Northwest China, Xinyu No. 18 parameters were: P5 = 820 °C·day, G2 = 920 kernels plant^−1^, G3 = 14.0 mg day^−1^. The extended P5 coefficient lengthened the grain-filling period while maintaining photoperiod sensitivity (PHINT unchanged). Cultivar switch simulations assumed 100% adoption with no yield penalty during transition years.

##### Combined Adaptation Strategy Cultivar Replacement

Integration of delayed planting by 5–15 days, appropriate supplemental irrigation, and late-maturing cultivar replacement, with optimal combinations tested per site and scenario to achieve maximum yield gains (Figure 2 and Figure 4). Combined treatments were designed to exploit synergies: delayed planting avoided peak heat, irrigation eliminated water deficits during critical stages, and late-maturing cultivars utilized the extended thermal time. Optimal combinations were identified through iterative testing of all permutations (3 planting dates × 2 irrigation levels × 2 cultivars = 12 combinations per site), selecting the highest-yielding scenario for each future period and RCP. Yield changes under each treatment and combination were reported as percentage differences relative to the baseline.

## 5. Conclusions

This cross-regional analysis demonstrates that future climate change under RCP4.5 and RCP8.5 will shorten maize growth periods (up to 34 days in Nakuru County, Kenya, and 38 days in Northwest China) and reduce yields (3–27% in Nakuru County; 5–22% in Northwest China) relative to the 1986–2005 baseline, primarily due to accelerated phenology from warming and stress during reproductive stages (Figure 1 and Figure 3). Kenya’s fully rainfed systems show greater sensitivity and more uniform losses compared to Northwest China’s partially irrigated sites. Combined adaptation measures—adjusting planting dates (delayed by 5–15 days), applying supplemental irrigation, and using late-maturing cultivars—can offset these declines and increase yields by up to ~39% in Nakuru County and ~29% in Northwest China (Figure 2 and Figure 4), with integrated approaches outperforming single measures and exhibiting broadly similar directional benefits across regions despite differences in magnitude.

Key limitations include reliance on a single GCM (HadGEM2-ES) with potential biases in precipitation and extremes, exclusion of pests, diseases, extreme events, economic factors, and adoption barriers, and assumptions of fixed soils and management—potentially inflating upper-end adaptation gains and reducing certainty in exact outcomes under real-world conditions. These findings provide biophysical evidence for prioritizing multi-strategy adaptations (optimized planting dates, irrigation expansion, and climate-resilient cultivars) in agricultural policies and extension programs for vulnerable maize systems. Policymakers should prioritize heat avoidance strategies in agricultural investment plans, mandate irrigation efficiency upgrades (drip/micro-sprinkler) at water-stressed sites, and implement climate-informed water allocation planning. Extension services should deploy revised planting calendars with 10–15 day delays, establish cultivar demonstration plots for H6218 (Kenya) and Xinyu No. 18 (China), and provide risk management tools to build farmer capacity for climate adaptation. The effectiveness of these adaptation measures will ultimately depend on their practical implementation under local farming conditions. While delayed planting and improved cultivars are readily applicable in many production systems, expansion of supplemental irrigation should be guided by sustainable water resource management and energy efficiency considerations. Integrating climate-resilient cultivars, optimized planting schedules, and efficient irrigation practices provides a practical pathway for enhancing maize resilience under future climate change in both Kenya and Northwest China. Future research should integrate multi-model climate projections with socio-economic assessments of farmer adoption, irrigation feasibility, and climate-resilient breeding strategies to develop adaptation pathways that are both scientifically robust and practically implementable under future climate change.

## Data Availability

Publicly available datasets used in this study include climate projection data from the HadGEM2-ES model under RCP4.5 and RCP8.5 scenarios, which are accessible through the Earth System Grid Federation (ESGF) portal (https://esgf-node.llnl.gov/projects/esgf-llnl/; accessed on 15 March 2020), and soil datasets available from the Chinese Soil Scientific Database (http://vdb3.soil.csdb.cn; accessed on 15 March 2020) and the ISRIC World Soil Information database (https://data.isric.org; accessed on 15 March 2020). Station-level meteorological and agronomic data obtained from the China Meteorological Administration (CMA) and the Kenya Meteorological Department (KMD) are subject to institutional data-sharing agreements and are therefore not publicly available; these data may be accessed upon formal request and approval from the respective agencies. Derived datasets generated during the study, including calibrated genetic coefficients, processed climate inputs, and simulation outputs (maize phenology and yield projections), as well as crop management files used in the CERES-Maize simulations, are available from the corresponding authors upon reasonable request.

## Supplementary Materials

The following supporting information can be downloaded at: https://www.mdpi.com/article/10.3390/plants15172709/s1, Figure S1: Geographical distribution of Study Sites in Nakuru County, Kenya; Figure S2: Geographical distribution of Study Sites in Northwest China; Figure S3: Sketch of the Structure of CERES Crop Model; Table S1: Information for validating the model on cultivar type and planting year at each site in Kenya; Table S2: Information for validating the model on cultivar type and planting year at each site in Northwest China; Table S3: Definition of genetic coefficients used in CERES-Maize (DSSAT v4.7) model; Table S4: Study sites projected changes in annual average temperature, precipitation and solar radiation relative to the baseline under RCP4.5 and RCP8.5; Table S5: Calculated genetic coefficients for the study sites maize cultivar; Figure S4: The phenological from sowing to flowering for maize (days) under future climate scenarios compared with six baseline stations in Kenya; Figure S5: The phenological from sowing to maturity (days) under future climate scenarios compared with six baseline stations in Kenya; Figure S6: Maize yield on the changed planting dates under RCP4.5 (2030s) compared to the future maize yield without adaptation measures; Figure S7: Maize yield on the changed planting dates under RCP4.5 (2050s) compared to the future maize yield without adaptation measures; Figure S8: Maize yield on the changed planting dates under RCP8.5 (2030s) compared to the future maize yield without adaptation measures; Figure S9: Maize yield on the changed planting dates under RCP8.5 (2050s) compared to the future maize yield without adaptation measures; Figure S10: Maize yield on the changed planting dates under RCP4.5 (2030s) under irrigation compared to the future maize yield without adaptation measures; Figure S11: Maize yield on the changed planting dates under RCP4.5 (2050s) under irrigation compared to the future maize yield without adaptation measures; Figure S12: Maize yield on the changed planting dates under RCP8.5 (2030s) under irrigation compared to the future maize yield without adaptation measures; Figure S13: Maize yield on the changed planting dates under RCP8.5 (2050s) under irrigation compared to the future maize yield without adaptation measures; Figure S14: Changes in maize yield (%) after cultivar change with H6218 under future climate scenarios based on one set of optimal treatment parameter compared with the future maize yield without adaptation measures for a-Njoro and b-Gilgil; Table S6: Study sites projected changes in annual average temperature, precipitation and solar radiation relative to the baseline under RCP4.5 and RCP8.5; Table S7: Calculated genetic coefficients for the study sites maize cultivar; Figure S15: The phenological from sowing to flowering for maize (days) under future climate scenarios compared with six baseline stations in Northwest China; Figure S16: The phenological from sowing to maturity (days) under future climate scenarios compared with six baseline stations in Northwest China; Figure S17: Maize yield on the changed planting dates under RCP4.5 (2030s) compared to the future maize yield without adaptation measures; Figure S18: Maize yield on the changed planting dates under RCP4.5 (2050s) compared to the future maize yield without adaptation measures; Figure S19: Maize yield on the changed planting dates under RCP8.5 (2030s) compared to the future maize yield without adaptation measures; Figure S20: Maize yield on the changed planting dates under RCP8.5 (2050s) compared to the future maize yield without adaptation measures; Figure S21: Maize yield on the changed planting dates under RCP4.5 (2030s) under irrigation compared to the future maize yield without adaptation measures; Figure S22: Maize yield on the changed planting dates under RCP4.5 (2050s) under irrigation compared to the future maize yield without adaptation measures; Figure S23: Maize yield on the changed planting dates under RCP8.5 (2030s) under irrigation compared to the future maize yield without adaptation measures; Figure S24: Maize yield on the changed planting dates under RCP8.5 (2050s) under irrigation compared to the future maize yield without adaptation measures; Figure S25: Changes in maize yield (%) after cultivar change with Xinyu No.18 under future climate scenarios based on one set of optimal treatment parameter compared with the future maize yield without adaptation measures for a-Hami and b-Jingyuan.

## Abbreviations

The following abbreviations are used in this manuscript:

AMESs	Agricultural Meteorological Experimental Stations
AR5	Fifth Assessment Report of the Intergovernmental Panel on Climate Change
CERES	Crop Environment Resource Synthesis
CIDP	County Integrated Development Plan
CMA	China Meteorological Administration
CMIP5	Coupled Model Intercomparison Project Phase 5
DSSAT	Decision Support System for Agrotechnology Transfer
ESGF	Earth System Grid Federation
GCM	General Circulation Model
GLUE	Generalized Likelihood Uncertainty Estimation
IPCC	Intergovernmental Panel on Climate Change
ISRIC	International Soil Reference and Information Centre
KMD	Kenya Meteorological Department
KNBS	Kenya National Bureau of Statistics
NWC	Northwest China
RCP	Representative Concentration Pathway
UNFCCC	United Nations Framework Convention on Climate Change
