# Supplementary material for: Impacts of Climate Change and Adaptation Strategies on Maize Yield in Kenya and Northwest China
Source: Plants (Basel). 2026 Sep 3;15(17):2709. doi: 10.3390/plants15172709 (PMC13567471; doi:10.3390/plants15172709)
Supplement: Supplementary file 1 [file plants-15-02709-s001.zip › plants-4434873-supplementary-send conversion.pdf]

Supplementary Information for

Impact of Future Climate Change on Maize Yield and Contributions of Adaptation Measures on Yield Alleviation Change: Comparison in Similar Typical Area of Kenya and Northwest China.

Jackson K. Koimbori[a], Shuai Wang[b], Jie Pan[a], Liping Guo[a,*], Kuo Li[a,*]

[a]*Key Lab for Agro-Environment, Ministry of Agriculture and Rural Affairs, Institute of Environment and Sustainable Development in Agriculture, Chinese Academy of Agricultural Sciences, Beijing 100081, China;*
[b]*College of Land and Environment, Shenyang Agricultural University, Shenyang 110866, China;*
[c]*Department of Land Management, Zhejiang University, Hangzhou, Zhejiang 310058, China.*

.

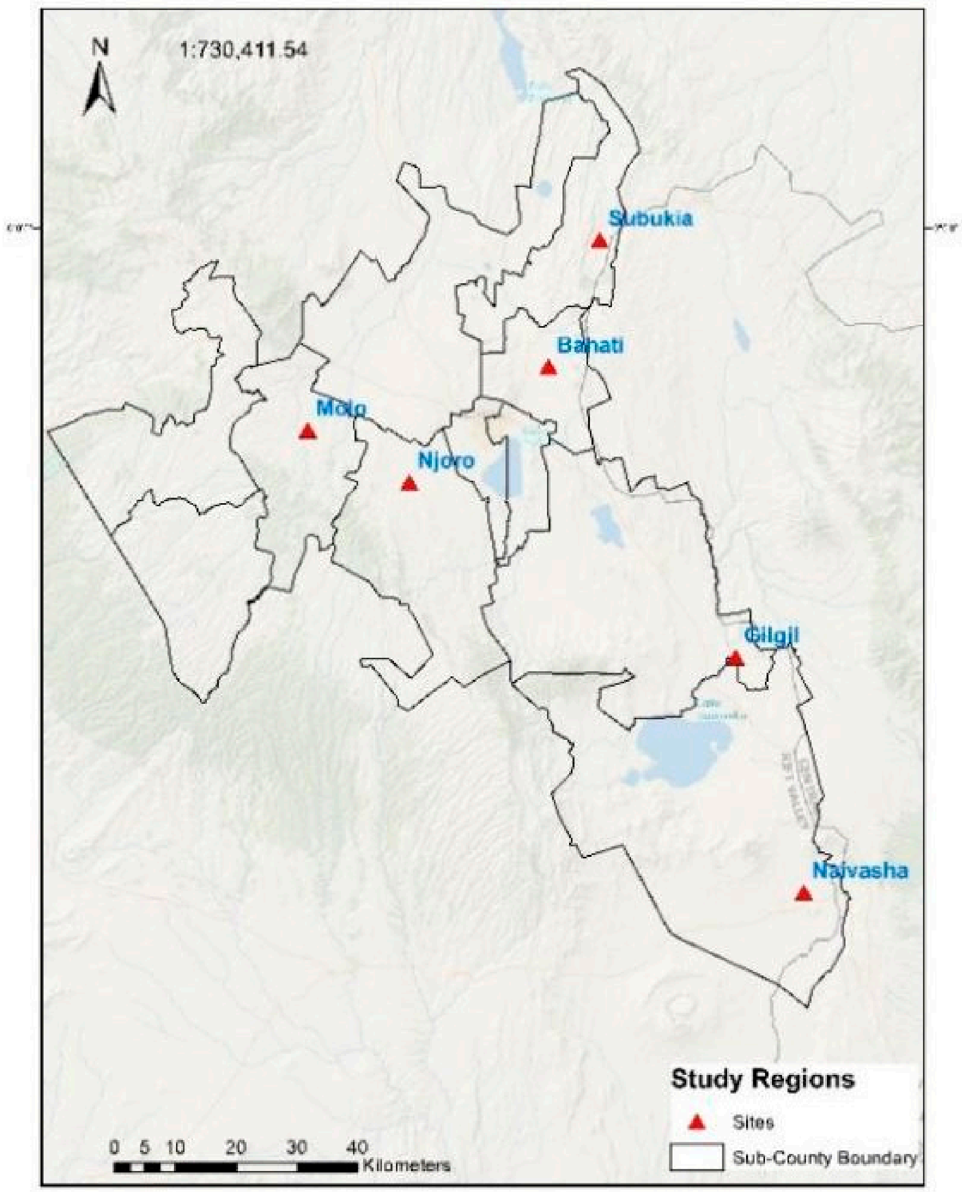

Fig. S1. Geographical distribution of Study Sites in Nakuru County, Kenya.

Source: Authors' own elaboration using GIS analysis based on shapefiles from [Kenya National Bureau of Statistics (KNBS) and GADM database]. © 2025 The Authors.

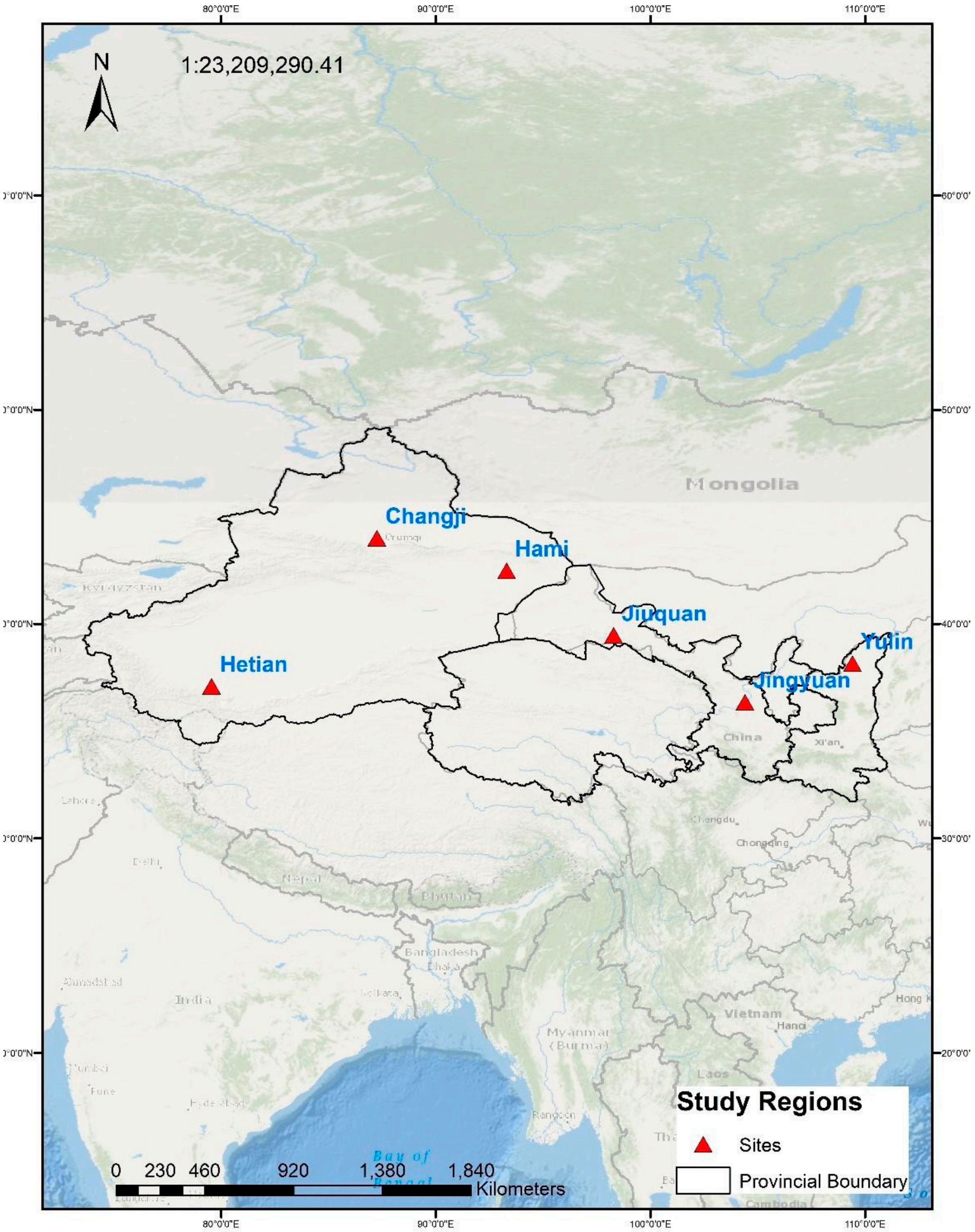

Fig. S2. Geographical distribution of Study Sites in Northwest China

Source: Authors' own elaboration using GIS analysis based on shapefiles from [National Geomatics Center of China (NGCC), and GADM database]. © 2025 The Authors.

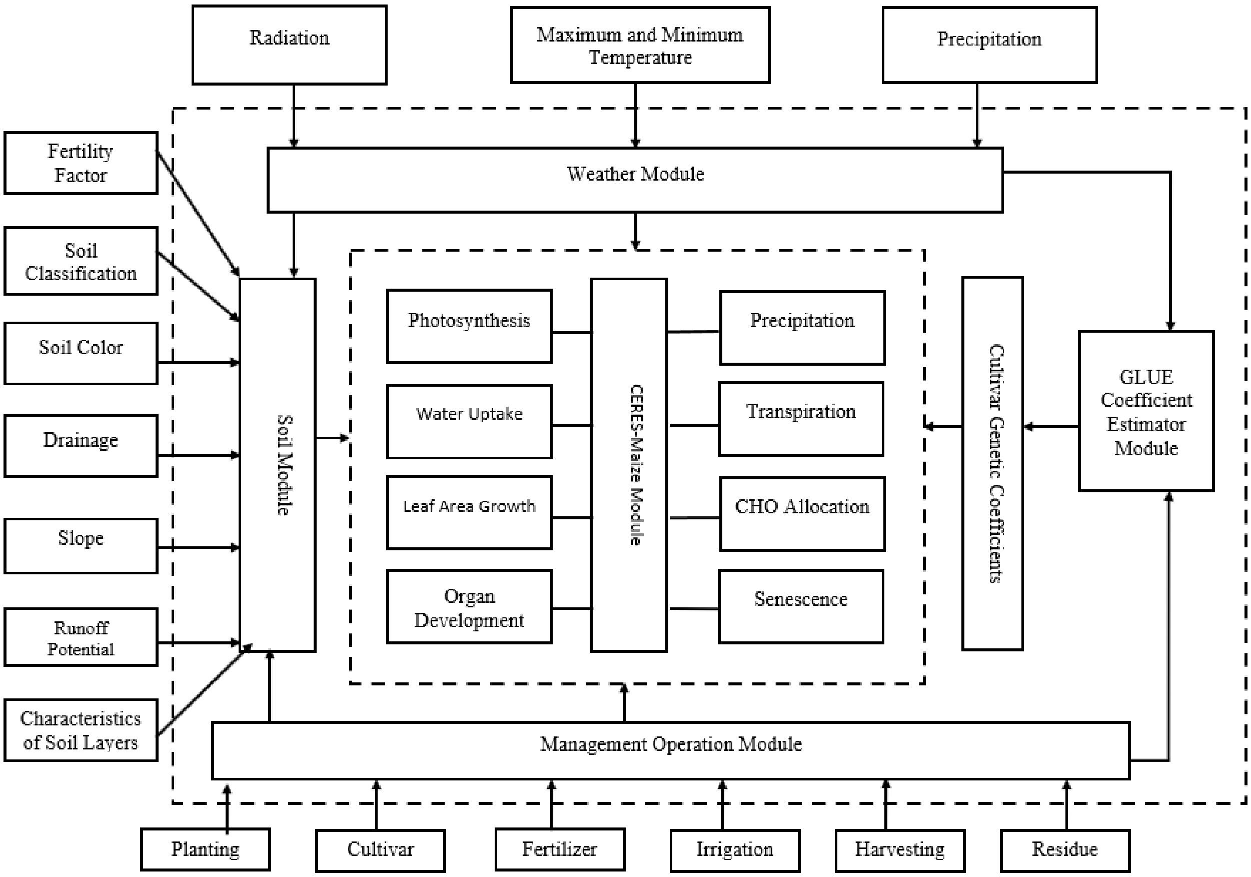

Fig. S3. Sketch of the Structure of CERES Crop Model

Table S1. Information for validating the model on cultivar type and planting year at each site in Kenya

**Province**	**Site**	**Latitude (N)**	**Longitude (E)**	**Altitude (m)**	**Maize Cultivar**	**Type of Maturity**	**Years**
Nakuru	Bahati	-0.21	36.15	2061	H6218	Late	2005,2006,2007*
	Gilgil	-0.63	36.43	1983	H513	Medium	2006,2008*,2009
	Molo	-0.38	35.8	2520	H6210	Late	2005,2006*,2008
	Naivasha	-0.98	36.53	1979	H520	Mid-late	2006*,2007,2008
	Njoro	-0.38	35.95	2227	H519	Medium	2006*, 2007, 2008
	Subukia	-0.02	36.23	2026	H624	Mid-late	2006,2007,2008*

* Indicates the years (observed records) used for calibration of the crop model.

Table S2. Information for validating the model on cultivar type and planting year at each site in Northwest China

**Province**	**Site**	**Latitude (N)**	**Longitude (E)**	**Altitude (m)**	**Maize Cultivar**	**Type of Maturity**	**Years**
Xinjiang	Hami	42.49	93.31	737.2	SC704	Medium	2006,2007,2008*
Xinjiang	Changji	44.01	87.26	577.5	Xinyu No.18	Mid-late	2007*,2008,2009
Xinjiang	Hetian	37.08	79.56	1375	Shen Yu 2002	Late maturity	2005*,2006,2009
Gansu	Jiuquan	39.46	98.29	1477.2	296	Medium	2005,2008,2009*
Gansu	Jingyuan	36.34	104.41	1398.2	Shendan No. 16	Medium	2005,2006,2008*
Shaanxi	Yulin	38.14	109.42	1057.5	Shendan No. 10	Medium	2005*/2006/2007

* Indicates the years (observed records) used for calibration of the crop model.

Table S3. Definition of genetic coefficients used in CERES-Maize (DSSAT v4.7) model.

**Coefficient**	**Definition**	**Unit**
P1	Thermal time from seedling emergence to the end of the juvenile phase (above a base temperature of 8°C) during which the plant is not responsive to changes in photoperiod	^{0}C days
P2	Extent to which development is delayed for each hour increase in photoperiod above the longest photoperiod at which development proceeds at a maximum rate (which is considered to be 12.5 h).	days
P5	Thermal time from silking to physiological maturity (expressed in degree days above a base temperature of 8°C).	^{0}C days
G2	Maximum possible number of kernels per plant.	Number
G3	Kernel filling rate during the linear grain filling stage and under optimum conditions.	Mg day^{-1}
PHINT	Phyllochron interval: the interval in thermal time between successive leaf tip appearances	^{0}C days

Table S4. Study sites projected changes in annual average temperature, precipitation and solar radiation relative to the baseline under RCP4.5 and RCP8.5.

**County**	**Study Site**	**Mean Temperature (°C)**			**Precipitation (mm) (%)**		**Solar Radiation (MJ m-2 d-1) (%)**	
Nakuru	Bahati	**Scenarios**	**2030s**	**2050s**	**2030s**	**2050s**	**2030s**	**2050s**
		RCP 4.5	0.68	0.8	4.68	7.2	-3.72	-6.54
		RCP 8.5	0.58	1.07	3.01	9.51	-3.72	1.74
	Gilgil	RCP 4.5	1.2	2.42	4.98	7.94	-1.52	-2.82
		RCP 8.5	1.14	2.69	3.52	10.15	-2.32	-5.35
	Molo	RCP 4.5	0.64	0.61	3.16	11.24	-1.24	-3.44
		RCP 8.5	0.71	2.35	5.55	8.73	-2.58	-6.43
	Naivasha	RCP 4.5	1.06	3.14	4.6	8.11	-4.89	-8.05
		RCP 8.5	0.89	3.35	4.06	6.07	-2.78	-6.28
	Njoro	RCP 4.5	1.28	2.37	6.65	11.76	-1.15	-3.51
		RCP 8.5	1.48	2.87	3.01	9.43	-1.97	-4.22
	Subukia	RCP 4.5	1.44	2.53	6.33	8.58	-4.15	-7.51
		RCP 8.5	1.42	2.85	3.28	9.35	-5.5	-7.84

Table S5. Calculated genetic coefficients for the study sites maize cultivar.

Calculated genetic coefficients of each maize cultivar									Flowering Period		Maturation Period		Maize Yield	
SITES	PARAMETER	CULTIVAR	P1	P2	P5	G2	G3	PHINT	NRMSE (%)	PD (%)	NRMSE (%)	PD (%)	NRMSE (%)	PD (%)
Bahati	1	H6218	196.9	1.304	962.6	986	16.01	49	2.5	3.4	2.2	-3.1	3.6	-1.7
Bahati	2	H6218	114.9	0.677	842.9	917.6	15.64	49	2.5	3.4	2.2	-3.1	4.1	-2.5
Bahati	3	H6218	203.4	0.473	832.5	900.5	15.28	49	3.6	3.4	3.1	-3.1	2.6	-2.9
Bahati	4	H6218	109.5	1.251	864.1	527.6	15.98	49	2.5	2.2	2.2	-3.1	4.2	2.9
Bahati	5	H6218	221.5	1.765	714.7	926	14.69	49	2.5	3.4	2.2	-3.1	2.6	-5.0
Bahati	6	H6218	217.3	0.519	835.3	945.5	15.52	49	2.5	3.4	2.2	-3.1	2.8	-7.4
Gilgil	1	H513	188.5	0.743	546.2	697.4	7.376	49	0.8	2.5	0.5	0.8	0.6	-1.9
Gilgil	2	H513	168.9	1.179	567.5	444.3	10.16	49	0.8	0.0	1.7	2.3	0.6	-1.8
Gilgil	3	H513	181.1	1.015	554.7	615.9	7.982	49	0.8	2.5	1.2	1.6	0.6	-1.9
Gilgil	4	H513	146.9	1.647	550.3	571.2	8.638	49	0.8	2.5	0.8	0.8	1.1	-0.9
Gilgil	5	H513	154.9	1.363	556	521.5	9.2	49	0.8	1.3	1.2	1.6	0.6	-1.9
Gilgil	6	H513	120.3	2.23	559.7	684.3	7.125	49	0.8	2.5	1.2	1.6	1.1	-0.8
Molo	1	H6210	164.5	0.084	476.3	803.4	6.526	49	1.1	2.2	1.2	-1.2	2.6	0.8
Molo	2	H6210	141.7	0.45	525.4	802	5.989	49	1.1	0.0	1.5	-0.6	3.1	-9.6
Molo	3	H6210	124.5	0.469	551.8	806.6	5.959	49	1.1	2.2	1.5	-1.2	4.0	-8.6
Molo	4	H6210	134.8	0.473	517.6	804.3	6.097	49	1.1	0.0	1.9	-0.6	2.7	-6.3
Molo	5	H6210	125.1	0.752	519.8	801.4	6.108	49	1.1	0.0	1.5	-0.6	2.0	-10.0
Molo	6	H6210	135.2	0.509	511.8	799.3	6.35	49	1.1	2.2	1.9	-1.2	3.6	0.4
Naivasha	1	H520	271.1	1.469	639.9	608	8.64	49	4.5	-2.5	1.5	-1.5	2.3	3.6
Naivasha	2	H520	270.5	1.675	579.2	878.3	11.99	49	4.5	0.0	1.5	-1.5	0.3	-0.2
Naivasha	3	H520	267.9	1.258	636	717.8	5.928	49	4.5	0.0	1.5	0.0	0.6	1.2
Naivasha	4	H520	282.5	1.377	607	835.1	12.72	49	5.8	-2.5	1.5	-2.3	0.8	1.5
Naivasha	5	H520	271.1	1.447	614.5	389.7	13.23	49	5.8	-2.5	1.5	-2.3	2.4	2.0
Naivasha	6	H520	255.3	1.79	627.2	484.2	10.87	49	4.5	0.0	1.5	0.0	2.7	3.4
Njoro	1	H519	284.5	0.889	660.7	310.3	12.66	49	2.1	2.7	1.2	-2.2	5.6	-6.1
Njoro	2	H519	297.1	0.298	678.9	812.4	4.86	49	2.1	2.7	1.2	-2.2	4.4	-8.8
Njoro	3	H519	286.1	0.773	679.6	331.5	10.82	49	1.3	4.0	1.5	-0.7	4.0	-7.5
Njoro	4	H519	265.6	1.192	679.2	264.9	12.85	49	1.3	4.0	1.2	-3.0	4.7	-9.7
Njoro	5	H519	269.7	1.137	687.1	286.7	12.13	49	1.3	2.7	1.2	-3.0	4.4	-7.3
Njoro	6	H519	297.5	0.215	714.3	808.4	3.90	49	1.3	4.0	1.2	-0.7	4.8	-8.8
Subukia	1	H624	150.9	1.856	459	730.6	7.483	49	1.4	-1.5	0.8	0.8	7.0	8.7
Subukia	2	H624	192.8	0.446	446.6	736.3	8.187	49	1.4	-1.5	0.8	0.8	6.7	9.6
Subukia	3	H624	136.9	1.843	461.8	705.5	8.648	49	1.4	-2.9	0.8	0.8	8.6	7.4
Subukia	4	H624	181.4	0.831	481.9	695.1	7.975	49	1.4	-1.5	0.8	0.8	5.5	7.2
Subukia	5	H624	140.4	2.012	474.7	710.3	8.078	49	1.4	-2.9	0.8	0.8	9.0	7.8
Subukia	6	H624	161.5	1.466	476.5	705.7	8.146	49	1.4	-1.5	0.8	1.6	5.4	8.2

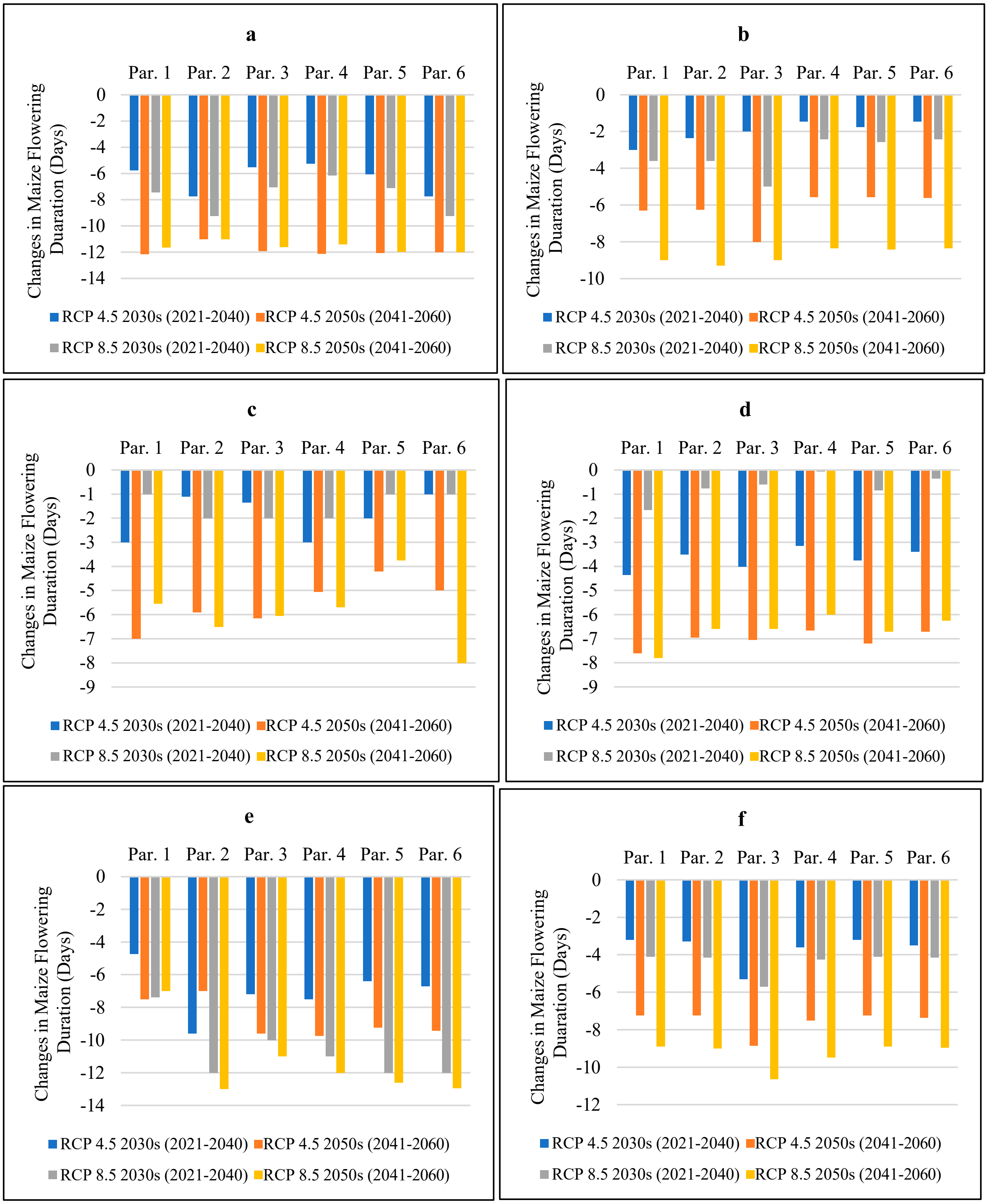

Fig. S4. The phenological from sowing to flowering for maize (days) under future climate scenarios compared with six baseline stations in Kenya.

(Note: Station a~f denotes Bahati, Gilgil, Molo, Naivasha, Njoro and Subukia, respectively)

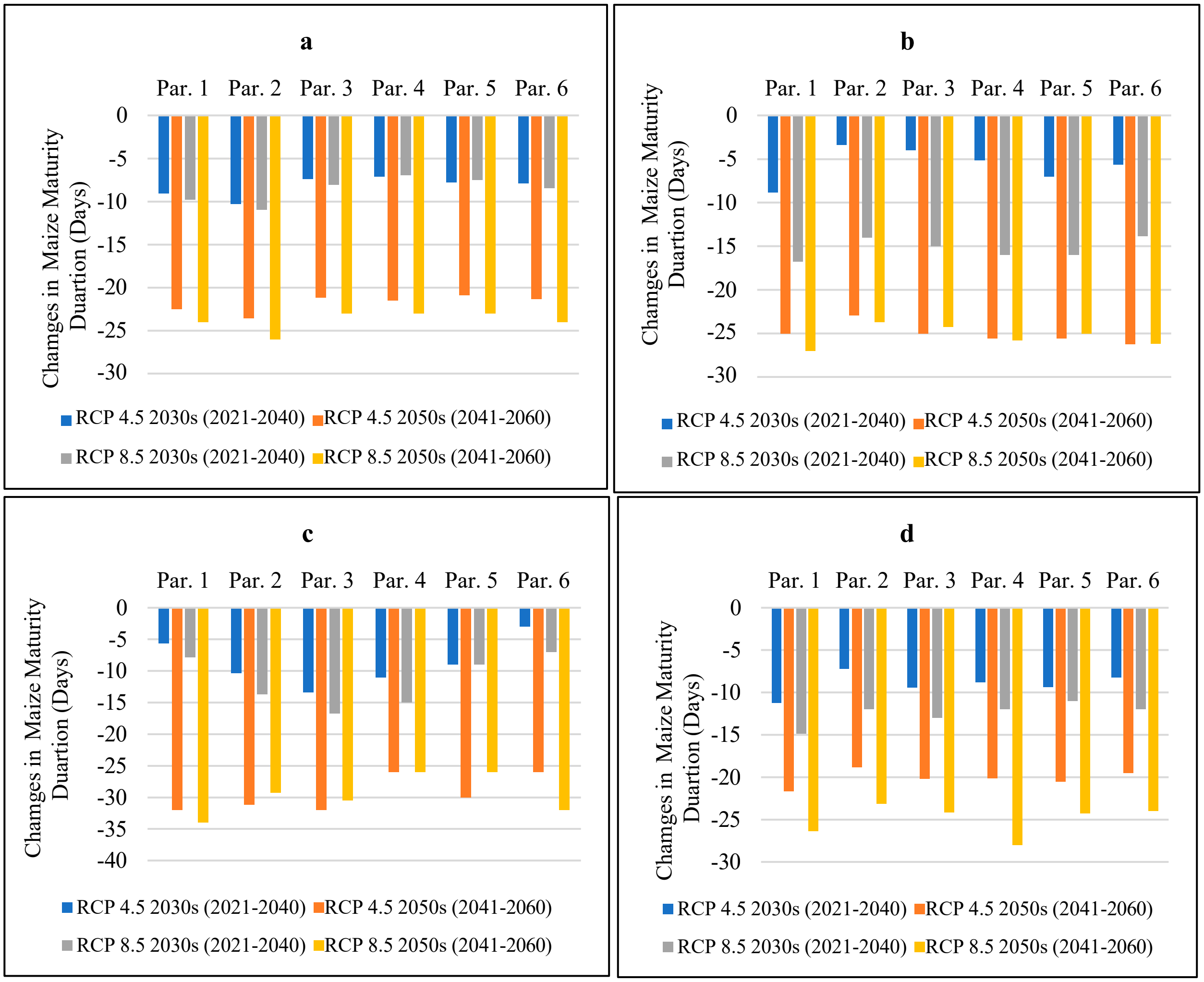

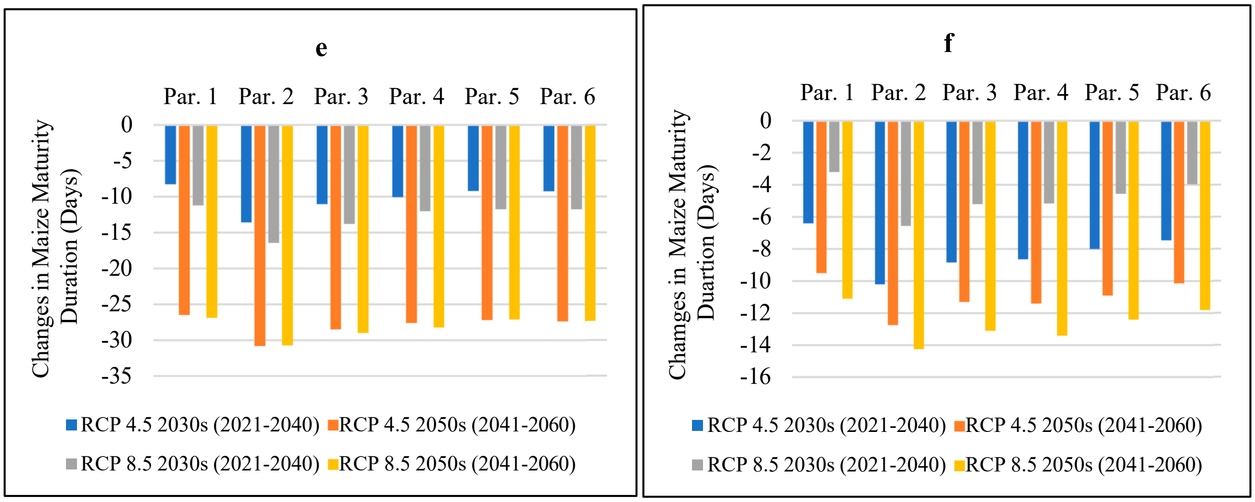

Fig. S5. The phenological from sowing to maturity (days) under future climate scenarios compared with six baseline stations in Kenya.

(Note: Station a~f denotes Bahati, Gilgil, Molo, Naivasha, Njoro and Subukia, respectively)

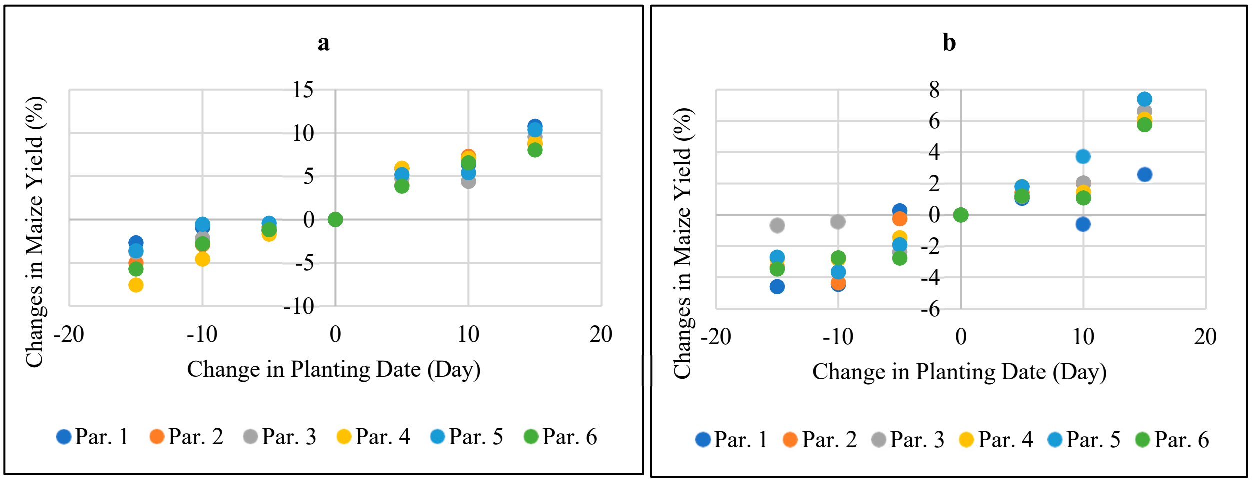

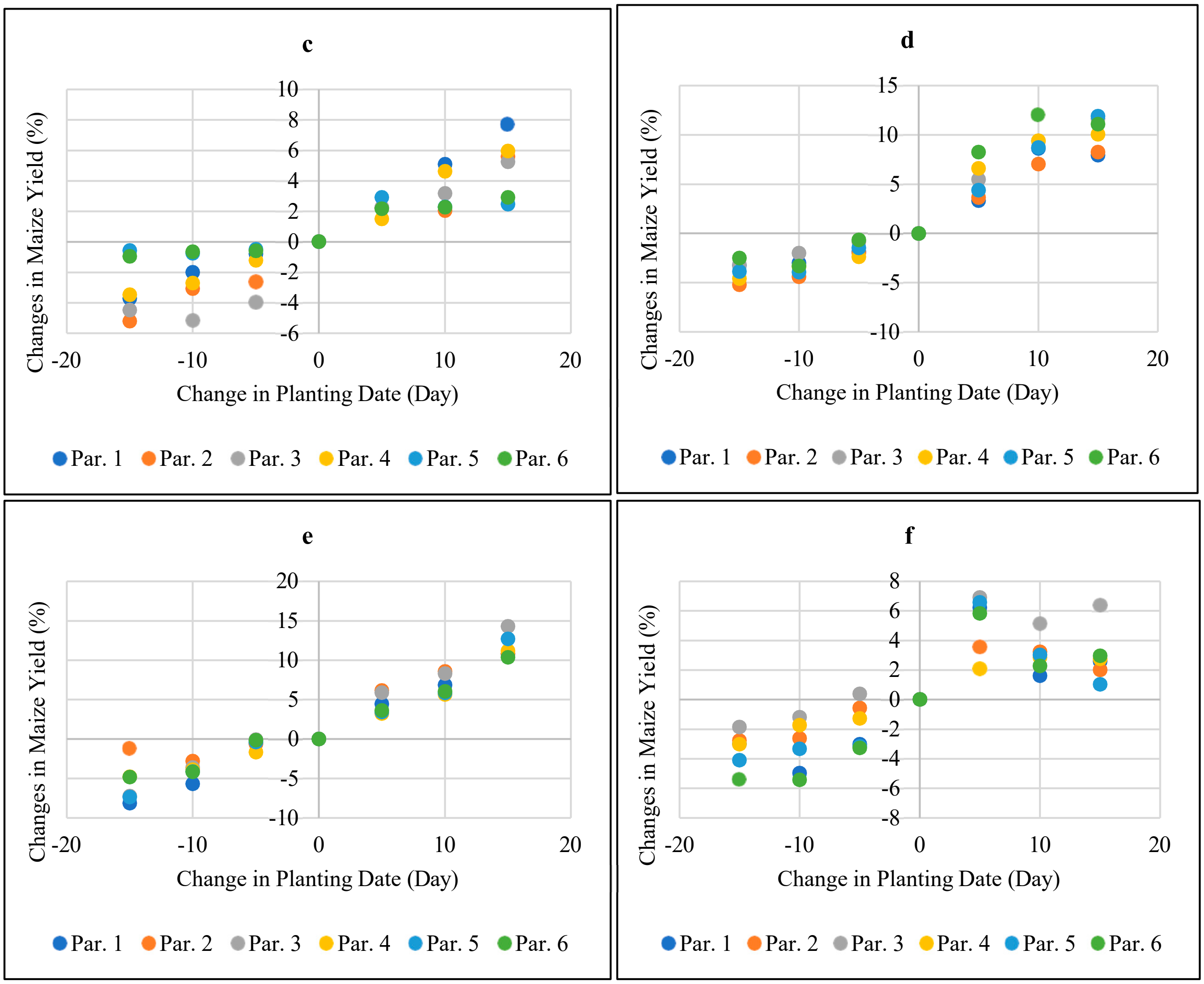

Fig. S6. Maize yield on the changed planting dates under RCP4.5 (2030s) compared to the future maize yield without adaptation measures.

(Note: Station a~f denotes Bahati, Gilgil, Molo, Naivasha, Njoro and Subukia, respectively)

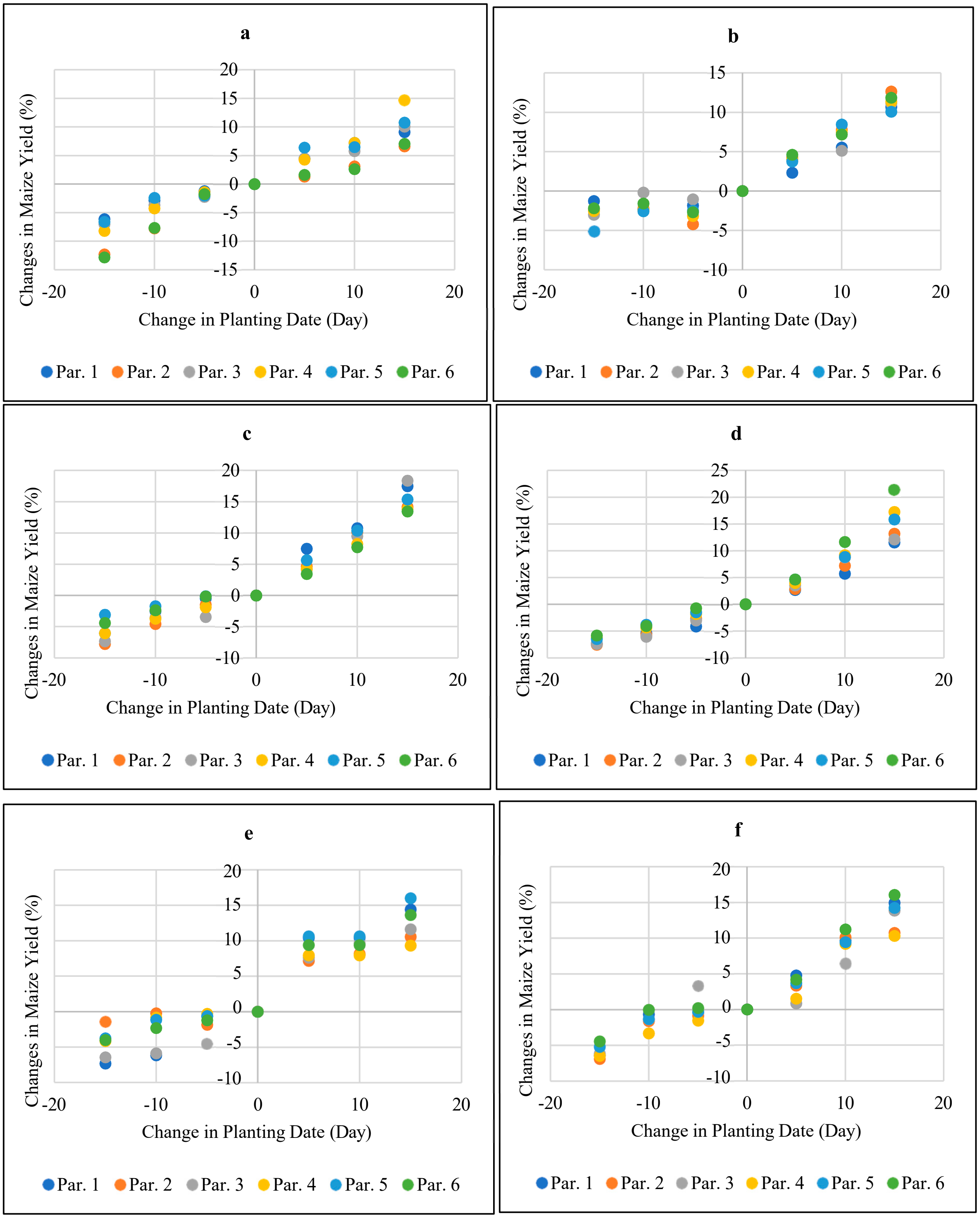

Fig. S7. Maize yield on the changed planting dates under RCP4.5 (2050s) compared to the future maize yield without adaptation measures.

(Note: Station a~f denotes Bahati, Gilgil, Molo, Naivasha, Njoro and Subukia, respectively)

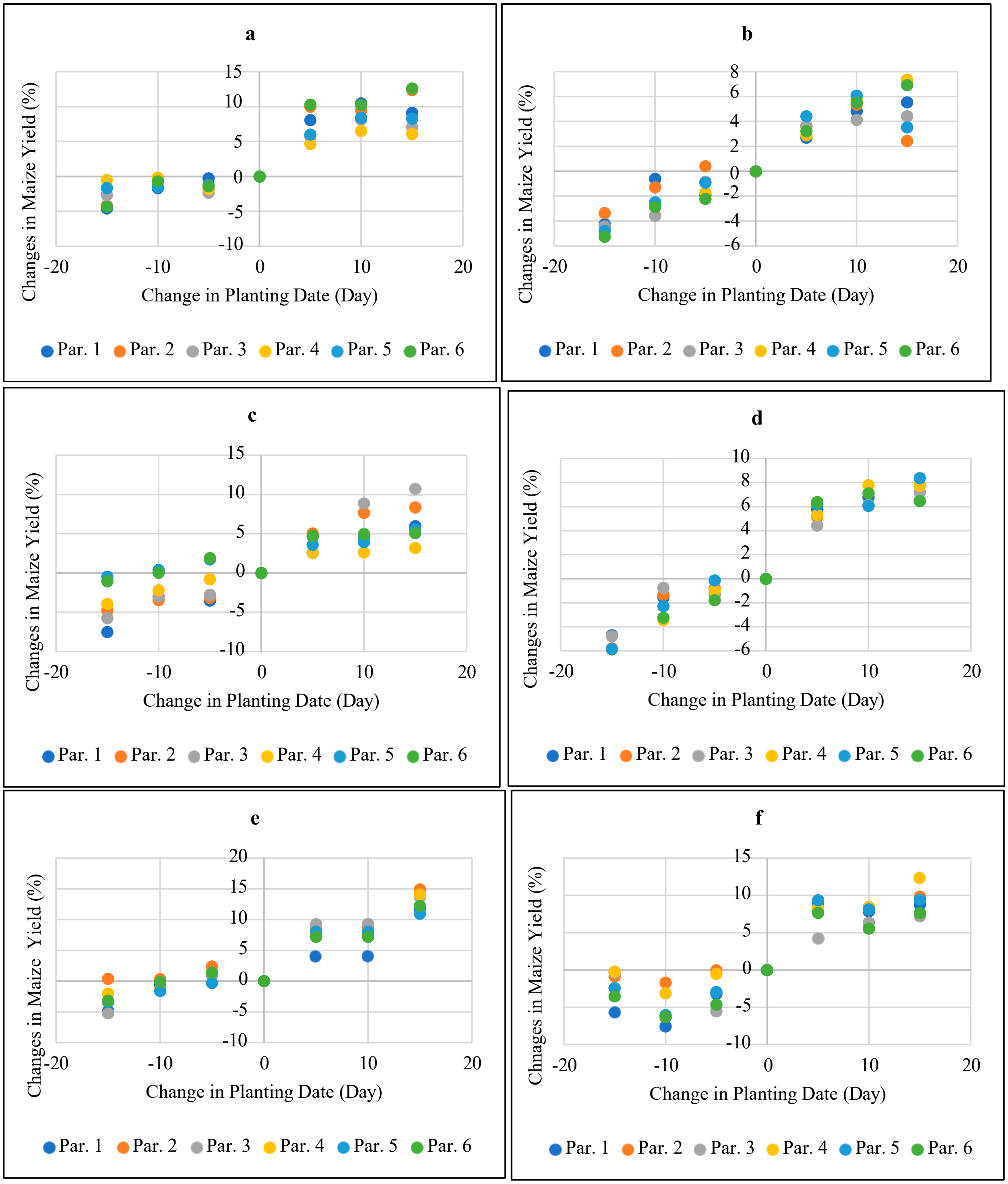

Fig. S8. Maize yield on the changed planting dates under RCP8.5 (2030s) compared to the future maize yield without adaptation measures.

(Note: Station a~f denotes Bahati, Gilgil, Molo, Naivasha, Njoro and Subukia, respectively)

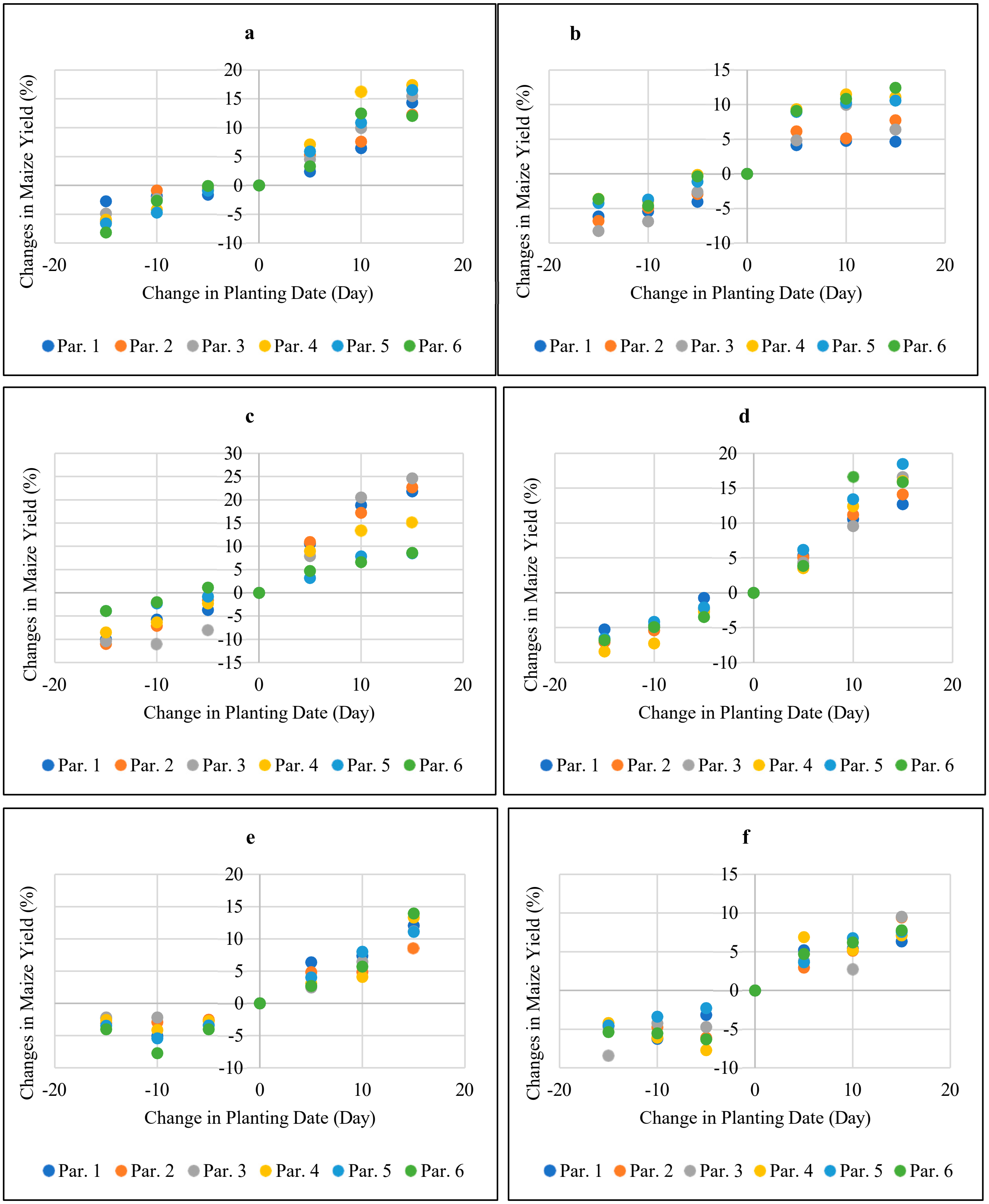

Fig. S9. Maize yield on the changed planting dates under RCP8.5 (2050s) compared to the future maize yield without adaptation measures.

(Note: Station a~f denotes Bahati, Gilgil, Molo, Naivasha, Njoro and Subukia, respectively)

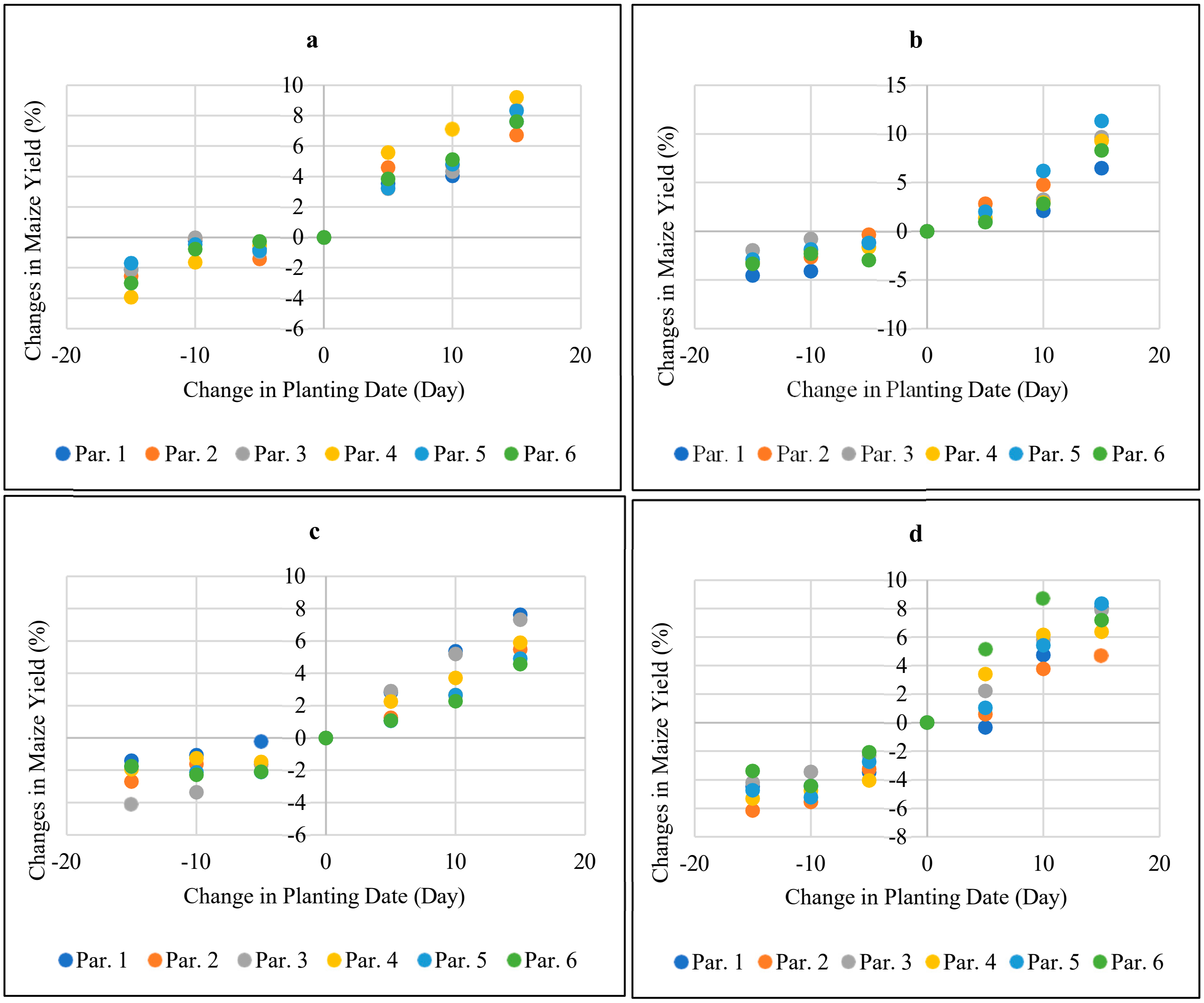

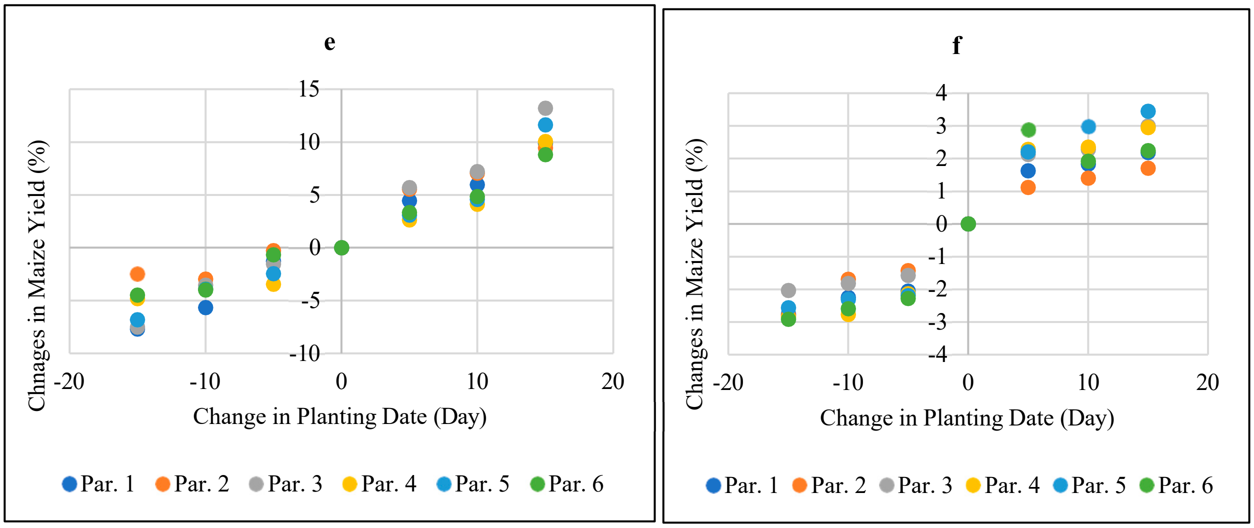

Fig. S10. Maize yield on the changed planting dates under RCP4.5 (2030s) under irrigation compared to the future maize yield without adaptation measures.

(Note: Station a~f denotes Bahati, Gilgil, Molo, Naivasha, Njoro and Subukia, respectively)

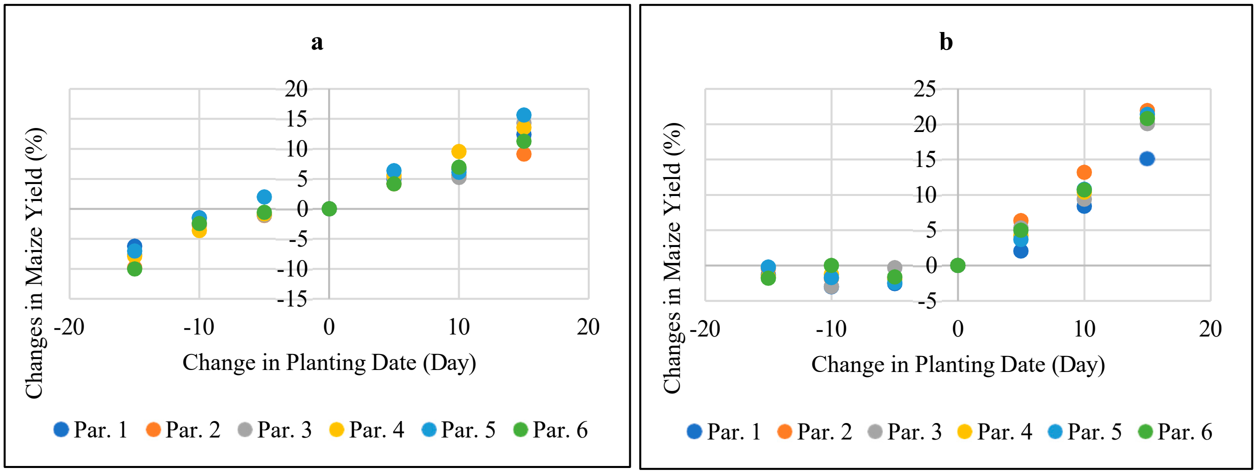

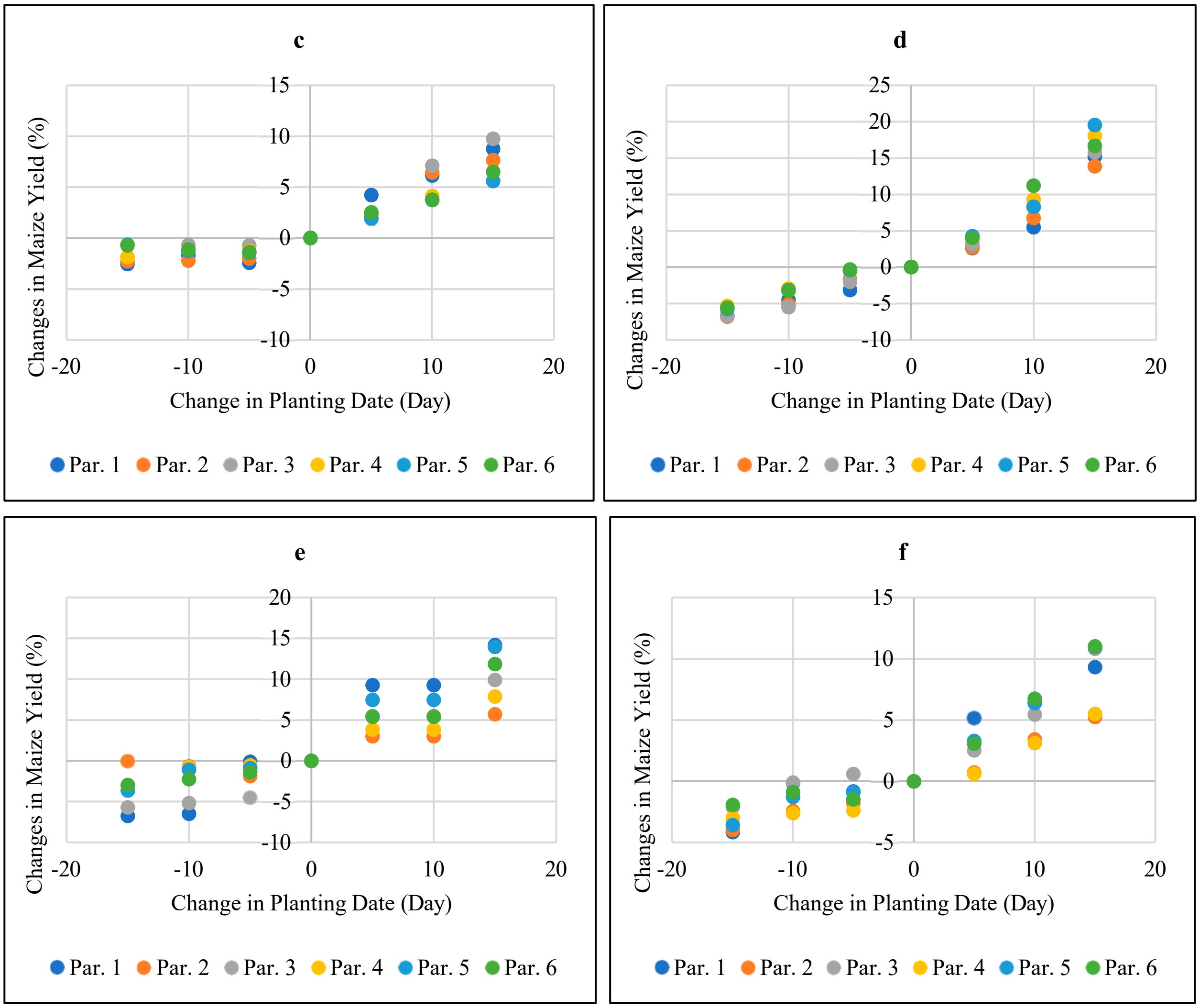

Fig. S11. Maize yield on the changed planting dates under RCP4.5 (2050s) under irrigation compared to the future maize yield without adaptation measures.

(Note: Station a~f denotes Bahati, Gilgil, Molo, Naivasha, Njoro and Subukia, respectively)

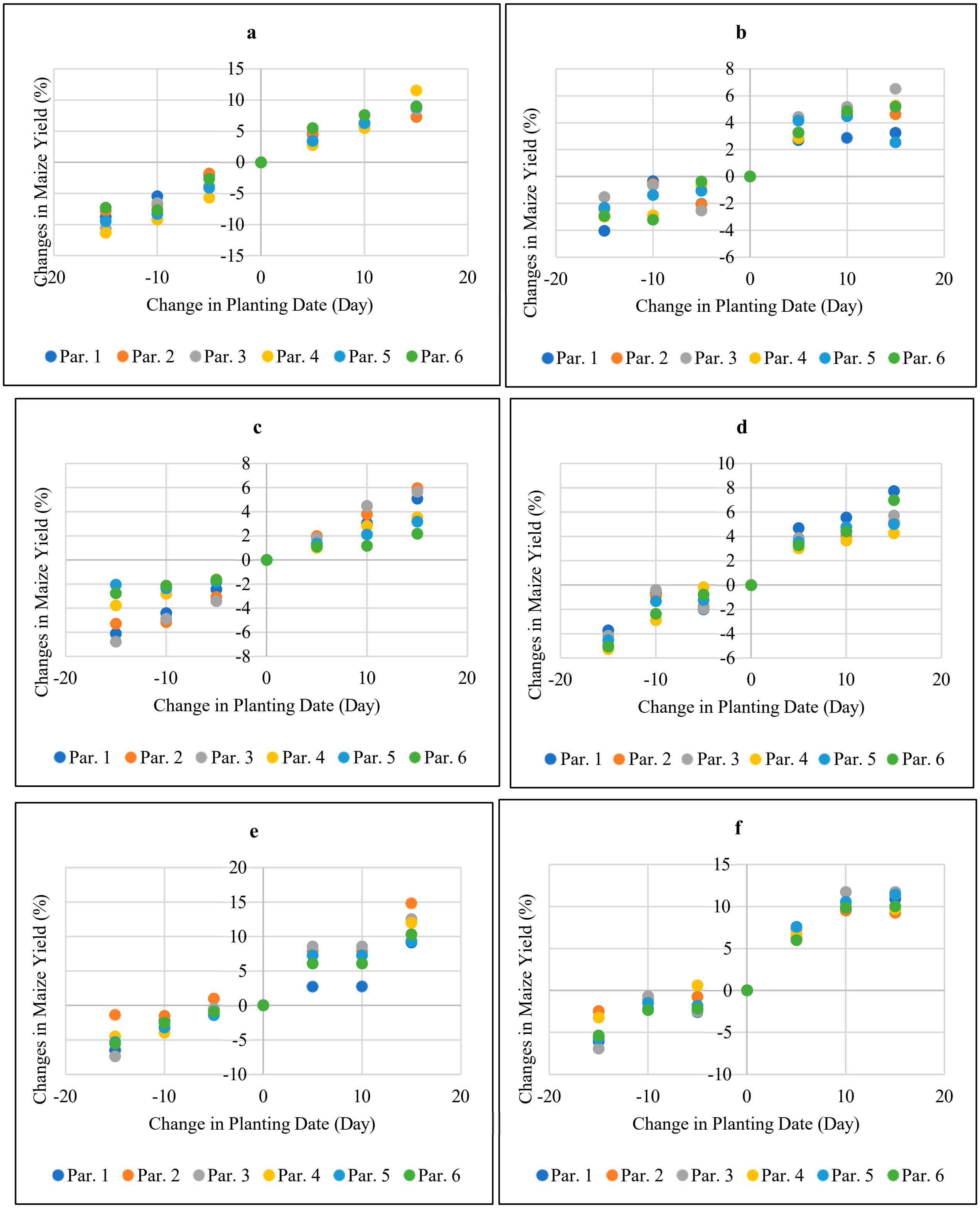

Fig. S12. Maize yield on the changed planting dates under RCP8.5 (2030s) under irrigation compared to the future maize yield without adaptation measures.

(Note: Station a~f denotes Bahati, Gilgil, Molo, Naivasha, Njoro and Subukia, respectively)

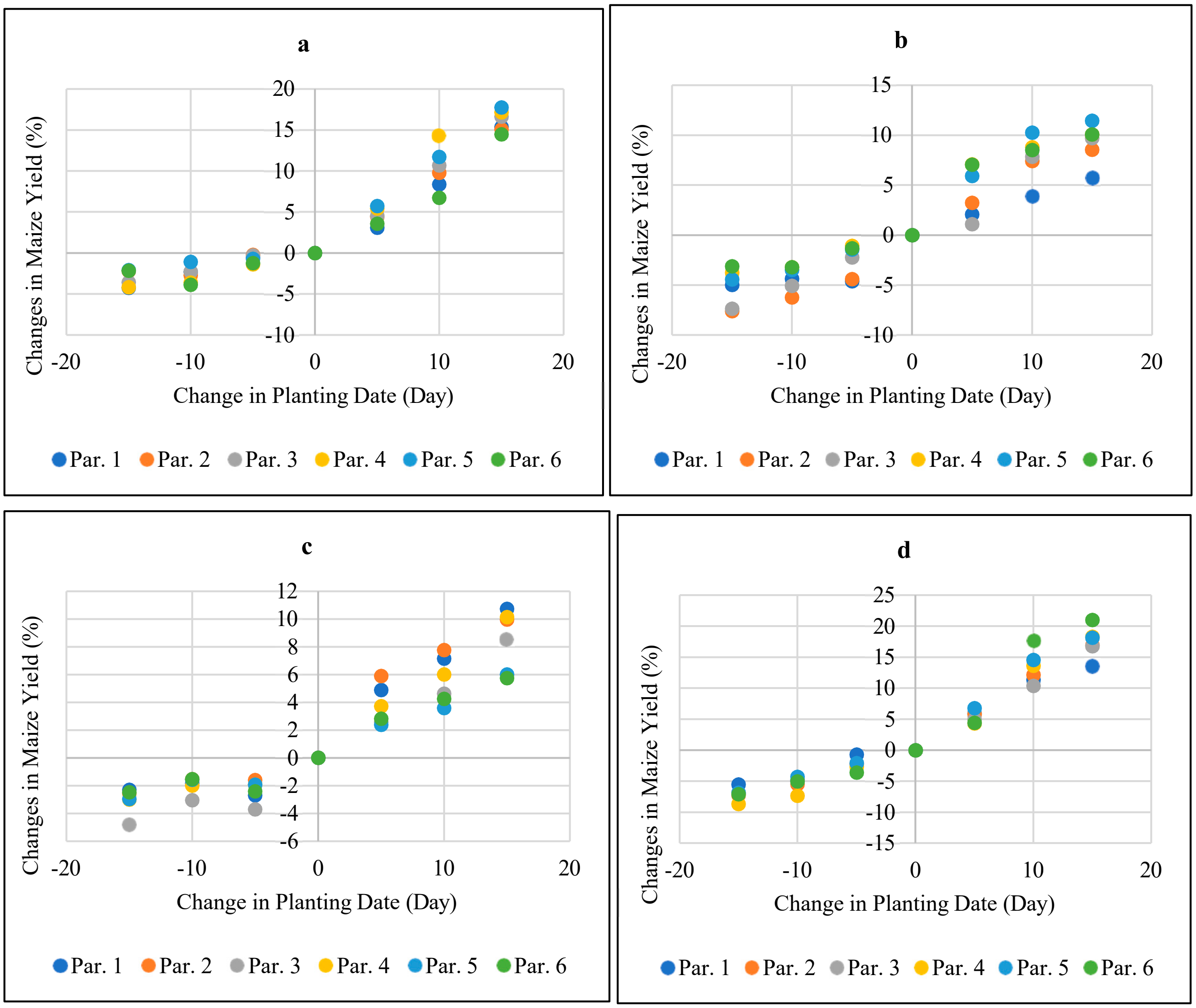

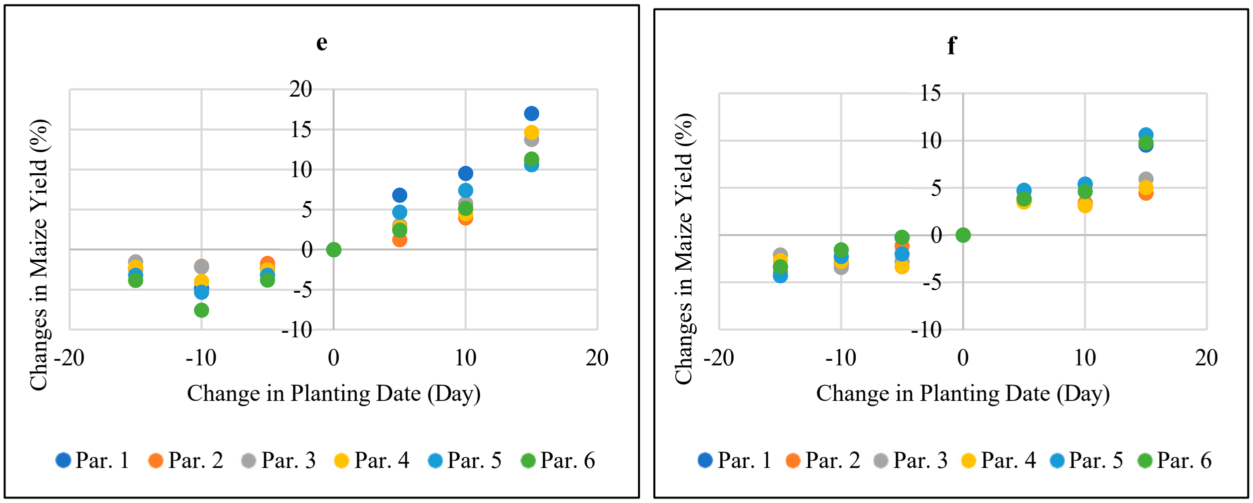

Fig. S13. Maize yield on the changed planting dates under RCP8.5 (2050s) under irrigation compared to the future maize yield without adaptation measures.

(Note: Station a~f denotes Bahati, Gilgil, Molo, Naivasha, Njoro and Subukia, respectively)

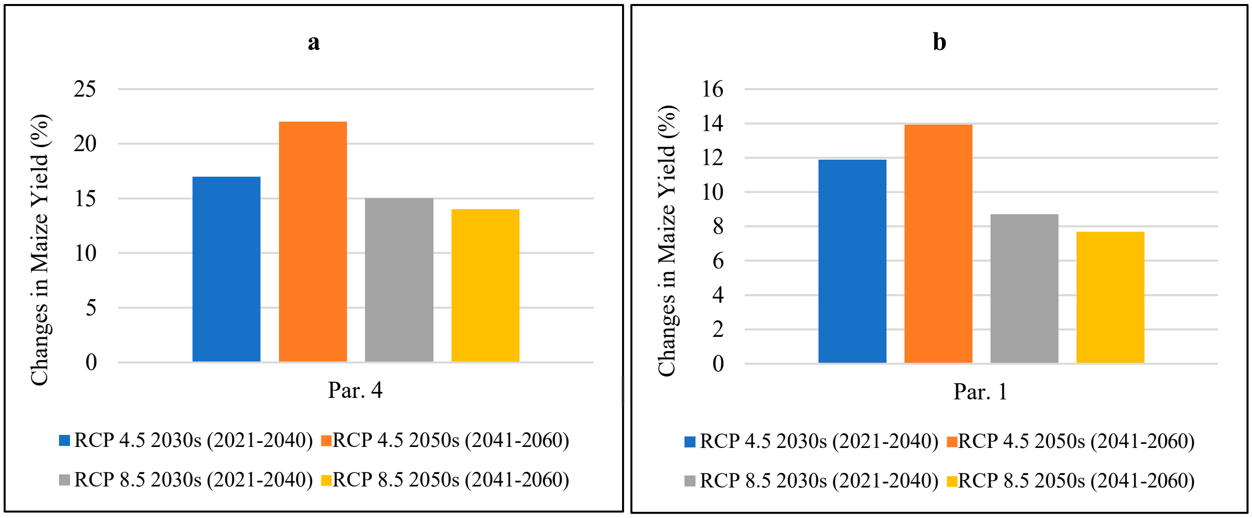

Fig. S14. Changes in maize yield (%) after cultivar change with H6218 under future climate scenarios based on one set of optimal treatment parameter compared with the future maize yield without adaptation measures for a-Njoro and b-Gilgil.

Table S6. Study sites projected changes in annual average temperature, precipitation and solar radiation relative to the baseline under RCP4.5 and RCP8.5.

**Province**	**Study Site**	**Mean Temperature (°C)**			**Precipitation (mm) (%)**		**Solar Radiation (MJ m-2 d-1) (%)**	
Xinjiang	Hami	**Scenarios**	**2030s**	**2050s**	**2030s**	**2050s**	**2030s**	**2050s**
		RCP 4.5	1.44	2.31	-0.35	6.07	-0.50	-0.73
		RCP 8.5	1.73	3.26	-2.34	15.55	-1.39	-1.36
Xinjiang	Changji	RCP 4.5	1.50	2.46	3.67	13.91	-0.55	-0.70
		RCP 8.5	0.48	1.93	11.19	12.30	-1.92	-1.36
Xinjiang	Hetian	RCP 4.5	1.48	2.28	24.96	39.49	-1.98	-2.29
		RCP 8.5	1.78	3.26	17.98	54.31	-3.00	-3.31
Gansu	Jiuquan	RCP 4.5	1.49	2.36	13.96	23.79	-1.19	-1.70
		RCP 8.5	1.74	3.19	38.83	31.93	-2.29	-2.42
Gansu	Jingyuan	RCP 4.5	1.02	1.77	12.66	13.40	-0.40	-0.53
		RCP 8.5	1.30	2.61	12.78	29.74	-0.81	-1.38
Shaanxi	Yulin	RCP 4.5	1.25	2.12	7.31	-2.69	-4.96	-3.30
		RCP 8.5	1.72	2.77	1.75	18.82	-4.87	-5.31

Table S7. Calculated genetic coefficients for the study sites maize cultivar

Calculated genetic coefficients of each maize cultivar									Flowering Period		Maturation Period		Maize Yield	
SITES	PARAMETER	CULTIVAR	P1	P2	P5	G2	G3	PHINT	NRMSE (%)	PD (%)	NRMSE (%)	PD (%)	NRMSE (%)	PD (%)
Hami	1	SC704	200	1.969	770.9	384.2	12.31	49	1	-2.7	1.2	-2.9	1.1	0.1
Hami	2	SC704	188.8	1.264	863	984.2	9.797	49	1	-2.7	1.2	-0.7	1.9	-1.7
Hami	3	SC704	189.8	1.267	870.9	947.3	11.32	49	2	1.3	2.1	1.4	0.8	-2.6
Hami	4	SC704	165.5	1.529	914.1	669.3	13.26	49	2	-1.3	2.1	-0.7	1.5	-2.2
Hami	5	SC704	173.6	1.505	977.2	931.6	10.26	49	2	-1.3	2.1	-0.7	1.1	-1.5
Hami	6	SC704	206.3	1.05	858.1	668.5	12.82	49	1	-2.7	1.2	1.4	2.2	-1.1
Changji	1	Xinyu No.18	281.9	1.615	740.2	531.8	15.17	49	3.6	-2.1	2.7	-3.3	5.9	-2.1
Changji	2	Xinyu No.18	274	1.832	746.7	583.5	14.88	49	3.6	-2.1	2.7	-3.3	1.3	-2.2
Changji	3	Xinyu No.18	296.8	1.458	628.4	561.9	15.08	49	3.6	-2.1	2.7	-3.3	2.7	-2.3
Changji	4	Xinyu No.18	282.2	1.726	774.3	484.4	16.12	49	3.6	-2.1	2.7	-3.3	4	-1.0
Changji	5	Xinyu No.18	278.1	1.883	926.8	555.1	15.22	49	3.6	-2.1	2.7	-3.3	3.8	-2.8
Changji	6	Xinyu No.18	297.3	1.376	623.9	515.8	15.71	49	3.6	-2.1	2.7	-3.3	5.7	-3.4
Hetian	1	Shen Yu 2002	185	1.719	670.9	264.2	9.97	49	1.3	-1.1	1.3	-1.5	4.1	1.9
Hetian	2	Shen Yu 2002	173.8	1.014	763	864.2	7.457	49	1.3	-2.2	1.3	-3.0	1.6	1.7
Hetian	3	Shen Yu 2002	174.8	1.017	770.9	827.3	8.98	49	1.3	-1.1	1.3	-2.2	0.6	-2.2
Hetian	4	Shen Yu 2002	150.5	1.279	814.1	549.3	10.92	49	0.9	-1.1	1.3	-1.5	1	-0.2
Hetian	5	Shen Yu 2002	158.6	1.255	877.2	811.6	7.92	49	0.9	-2.2	1.1	-1.5	1.2	1.3
Hetian	6	Shen Yu 2002	191.3	0.8	758.1	548.5	10.48	49	0.9	-1.1	1.1	-3.0	0.9	-0.3
Jiuquan	1	296	312.5	0.289	790	708.4	10.5	49	0.8	-3.0	0.4	0.0	1	-0.1
Jiuquan	2	296	311	0.323	818	711.2	11.65	49	0.8	-1.0	0.4	0.0	0.7	1.7
Jiuquan	3	296	301.3	0.218	801.2	714	12.8	49	0.8	1.0	0.4	1.2	0.5	1.3
Jiuquan	4	296	295.4	0.519	806.8	700	7.05	49	0	2.0	0.6	0.6	0.6	1.2
Jiuquan	5	296	300.2	0.233	812.4	702.8	8.2	49	0	-1.0	0.6	-1.8	0.3	0.7
Jiuquan	6	296	287.7	0.867	795.6	705.6	9.35	49	0.8	-3.0	0.6	-1.8	1	0.4
Jingyuan	1	Shendan No. 16	198.42	1.513	753.65	386.9	10.63	49	0.7	-2.2	1	-2.5	0.7	0.5
Jingyuan	2	Shendan No. 16	187.22	0.808	845.75	986.9	8.117	49	0.7	-2.2	1	-0.6	0.1	-0.8
Jingyuan	3	Shendan No. 16	188.22	0.811	853.65	950.0	9.64	49	0.7	1.1	1	1.3	1.1	-1.5
Jingyuan	4	Shendan No. 16	163.92	1.073	896.85	672.0	11.58	49	0.7	-1.1	1	-0.6	0.8	-1.2
Jingyuan	5	Shendan No. 16	172.02	1.049	959.95	934.3	8.58	49	0.7	-1.1	0.4	-0.6	0.4	-0.7
Jingyuan	6	Shendan No. 16	204.72	0.594	840.85	671.2	11.14	49	0.7	-2.2	0.4	1.3	0.4	-0.4
Yulin	1	Shendan No. 10	187	1.494	570.9	214.2	9.75	Yulin	1.1	-1.1	1.2	-0.7	4.4	-0.3
Yulin	2	Shendan No. 10	175.8	0.789	663	814.2	7.23	Yulin	3.2	-2.2	1.2	-2.2	1.7	-1.3
Yulin	3	Shendan No. 10	176.8	0.792	670.9	777.3	8.76	Yulin	1.1	-1.1	1.2	-1.5	0.7	-0.3
Yulin	4	Shendan No. 10	152.5	1.054	714.1	499.3	10.7	Yulin	1.1	-1.1	1.2	-0.7	1.1	-0.8
Yulin	5	Shendan No. 10	160.6	1.03	777.2	761.6	7.7	Yulin	1.1	-2.2	1.2	-0.7	1.3	-2.6
Yulin	6	Shendan No. 10	193.3	0.575	658.1	498.5	10.26	Yulin	1.1	-1.1	1.1	-2.2	1	2.0

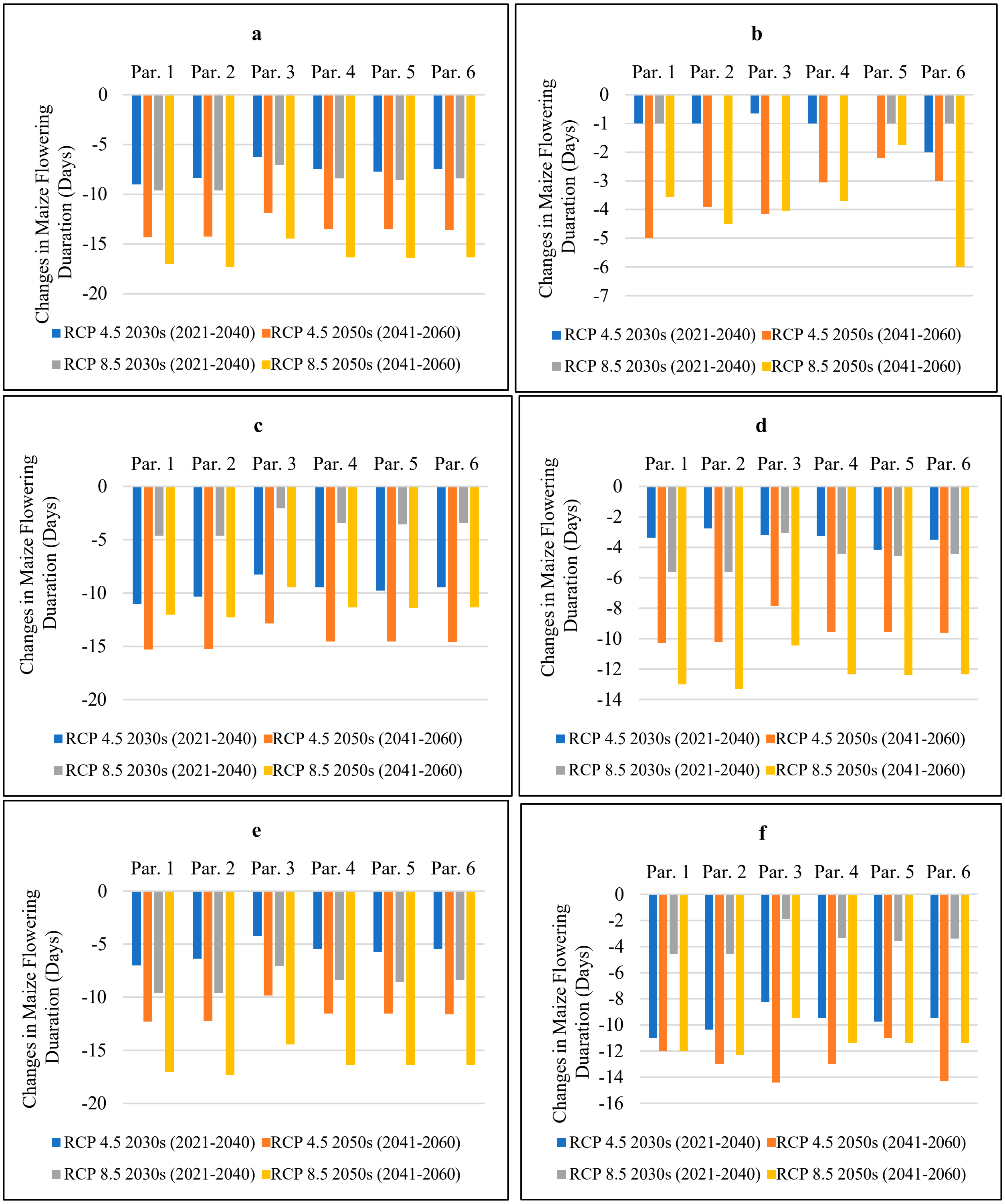

Fig. S15. The phenological from sowing to flowering for maize (days) under future climate scenarios compared with six baseline stations in Northwest China.

(Note: Station a~f denotes Hami, Changji, Hetian, Jiuquan, Jingyuan and Yulin, respectively)

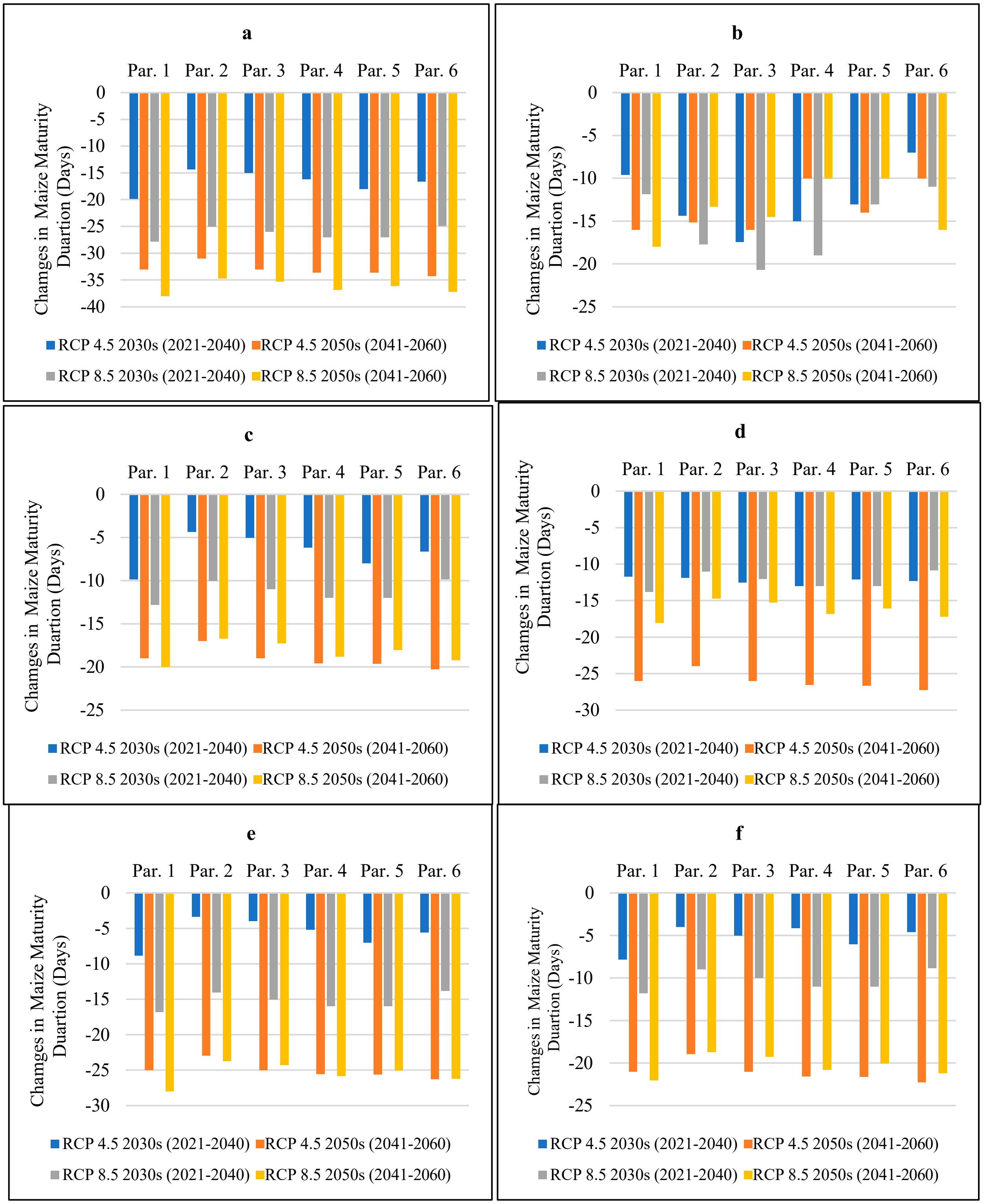

Fig. S16. The phenological from sowing to maturity (days) under future climate scenarios compared with six baseline stations in Northwest China.

(Note: Station a~f denotes Hami, Changji, Hetian, Jiuquan, Jingyuan and Yulin, respectively)

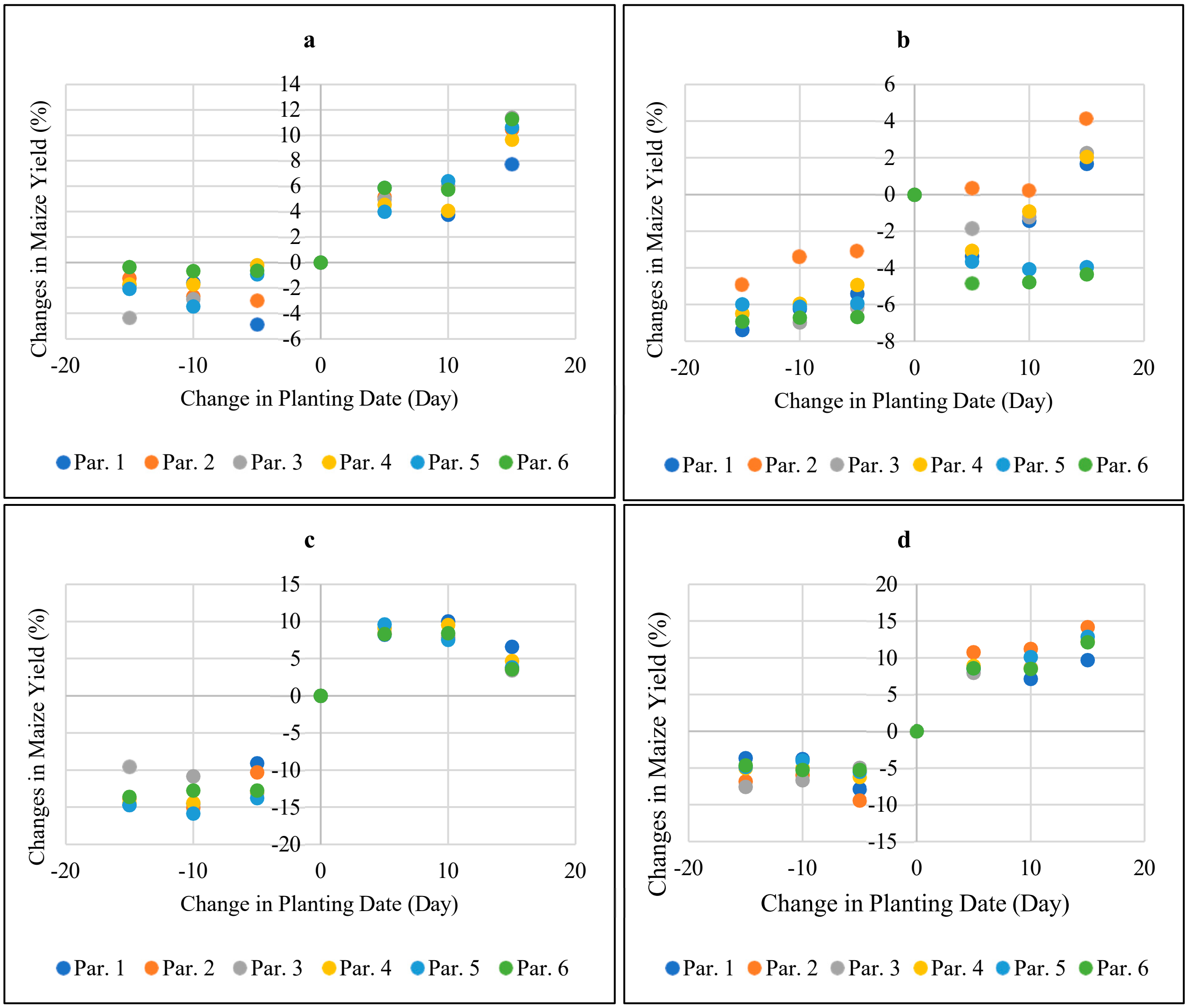

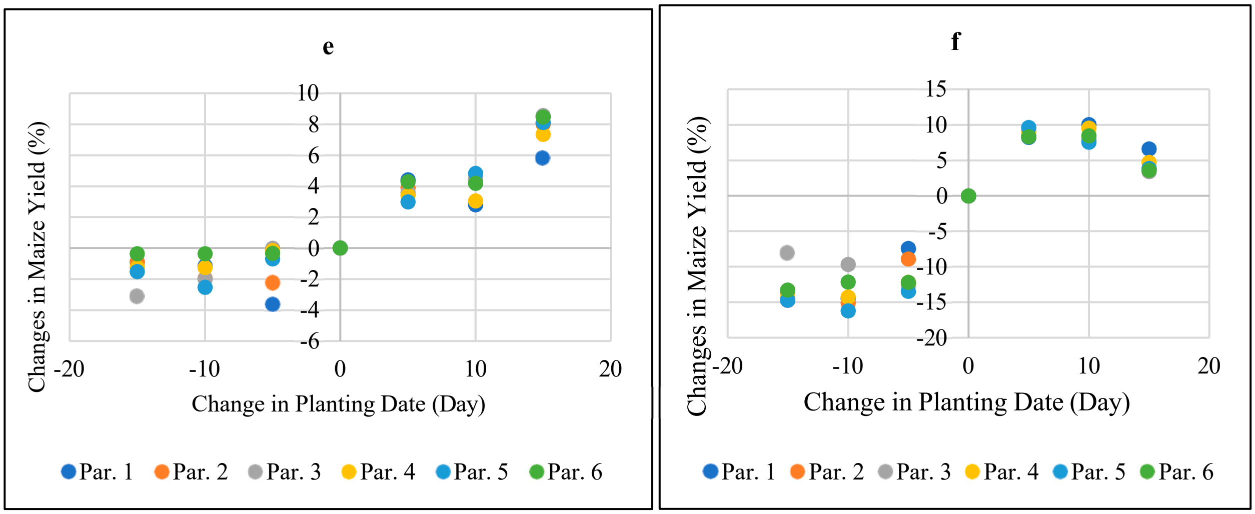

Fig. S17. Maize yield on the changed planting dates under RCP4.5 (2030s) compared to the future maize yield without adaptation measures.

(Note: Station a~f denotes Hami, Changji, Hetian, Jiuquan, Jingyuan and Yulin, respectively)

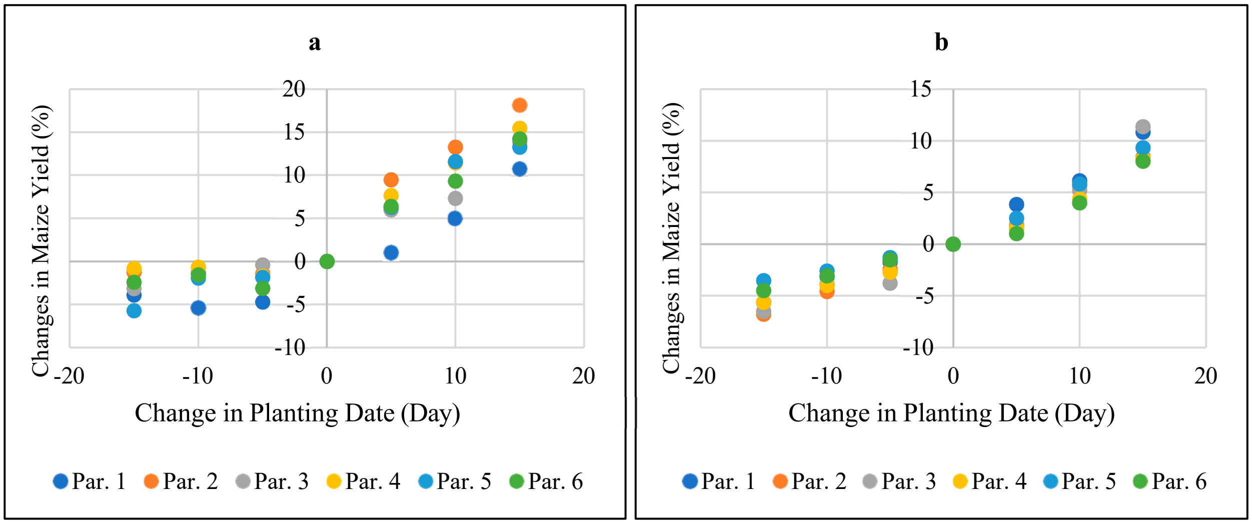

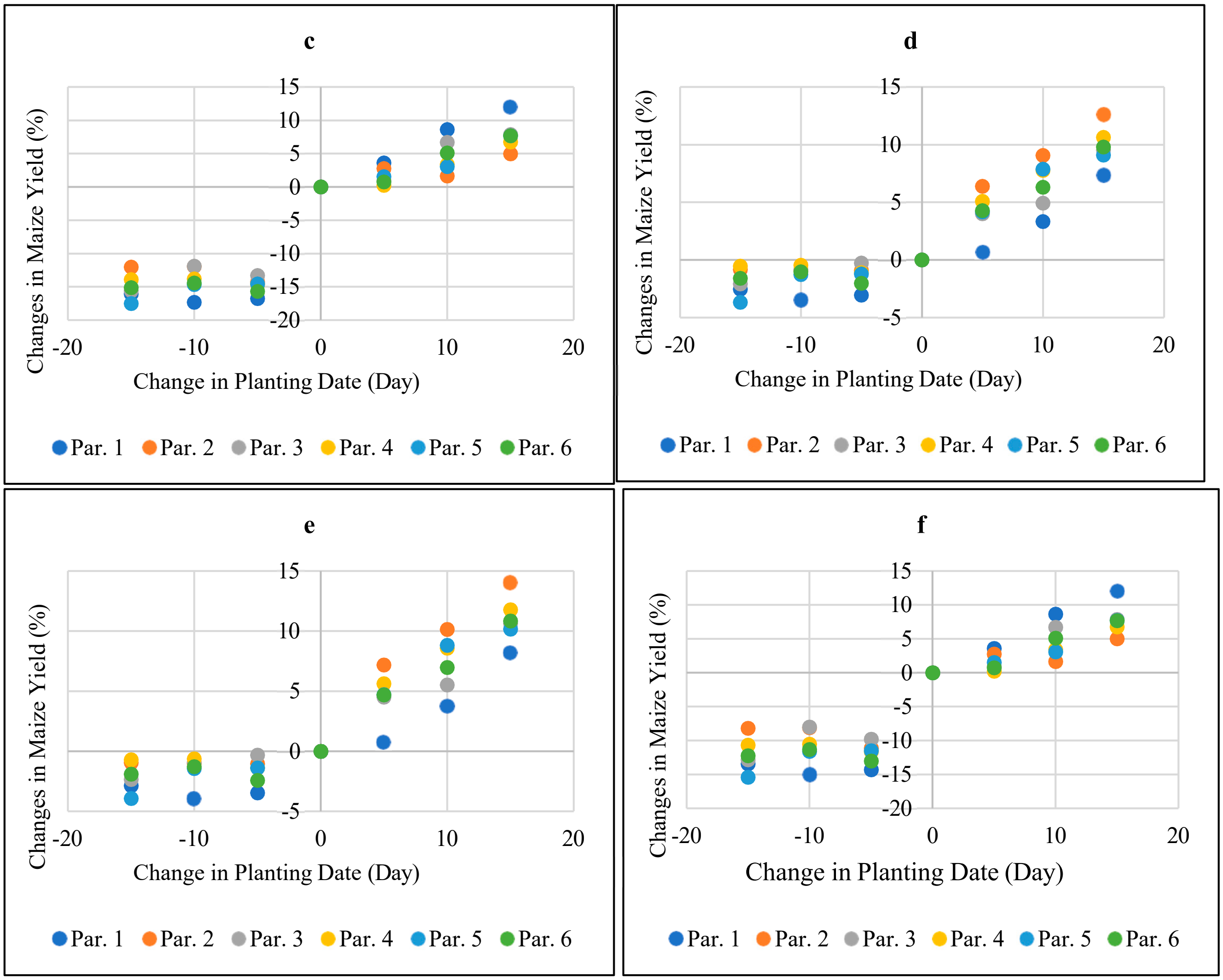

Fig. S18. Maize yield on the changed planting dates under RCP4.5 (2050s) compared to the future maize yield without adaptation measures.

(Note: Station a~f denotes Hami, Changji, Hetian, Jiuquan, Jingyuan and Yulin, respectively)

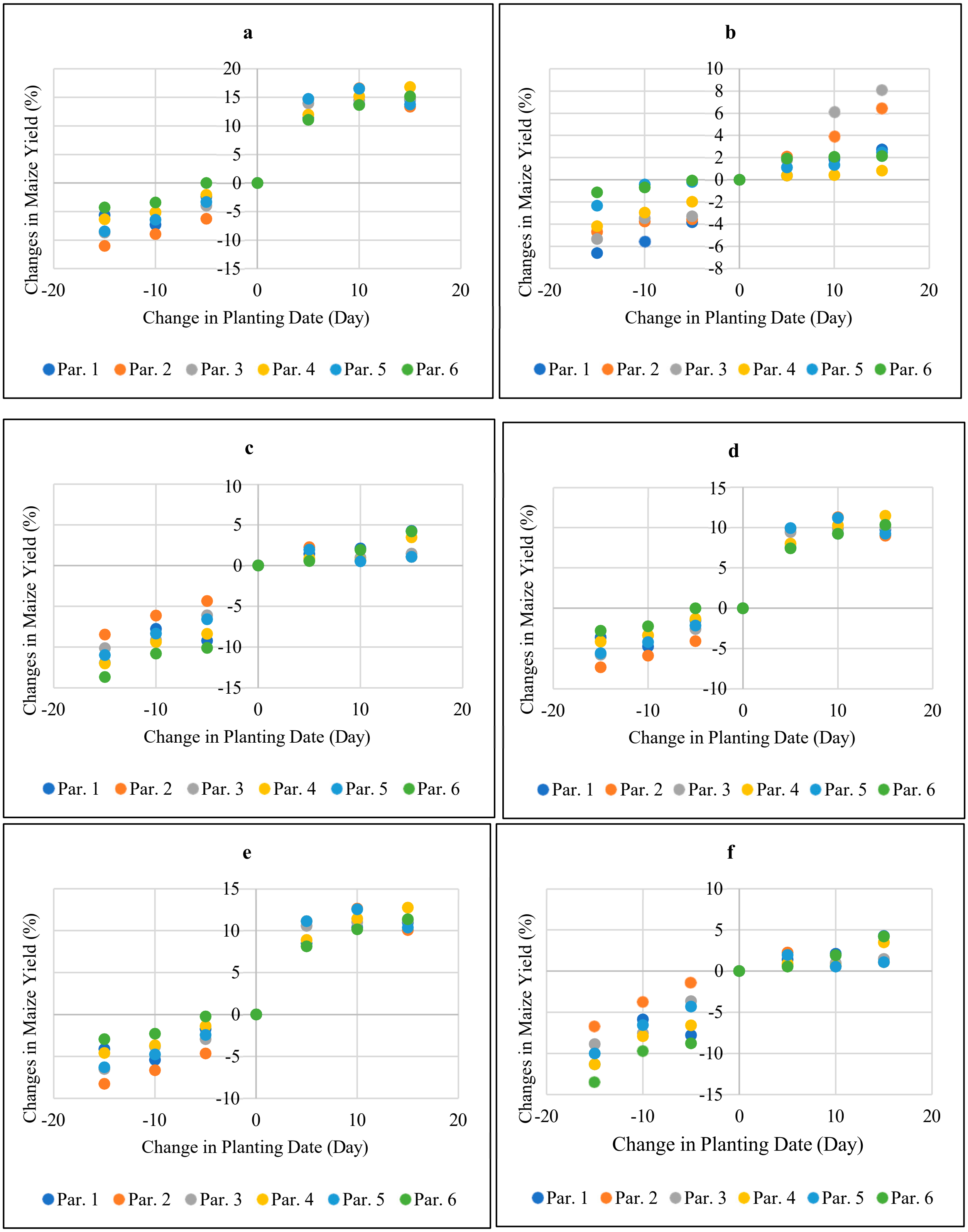

Fig. S19. Maize yield on the changed planting dates under RCP8.5 (2030s) compared to the future maize yield without

adaptation measures.

(Note: Station a~f denotes Hami, Changji, Hetian, Jiuquan, Jingyuan and Yulin, respectively)

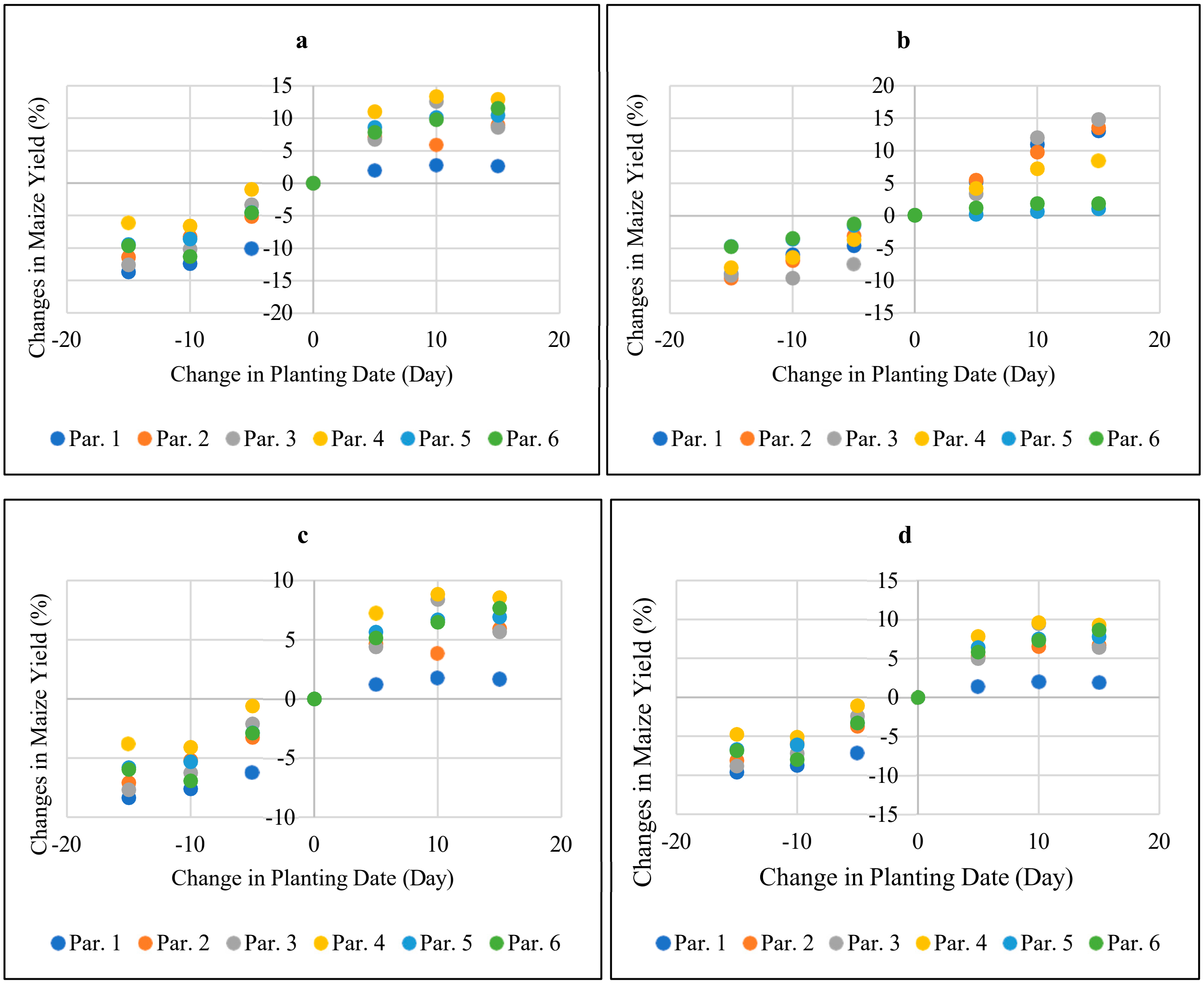

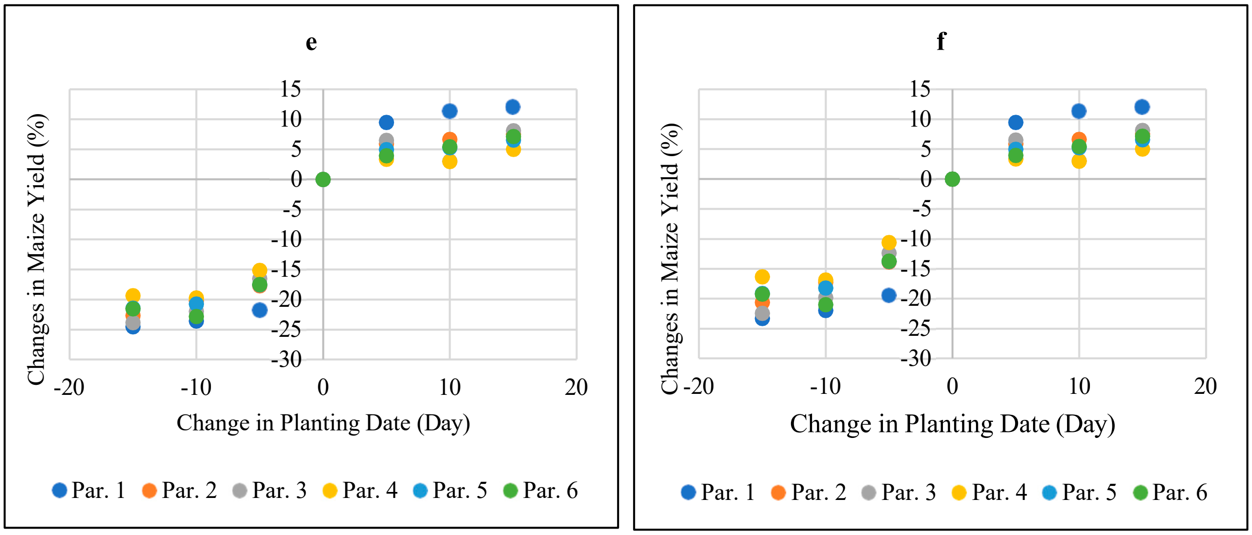

Fig. S20. Maize yield on the changed planting dates under RCP8.5 (2050s) compared to the future maize yield without adaptation measures.

(Note: Station a~f denotes Hami, Changji, Hetian, Jiuquan, Jingyuan and Yulin, respectively)

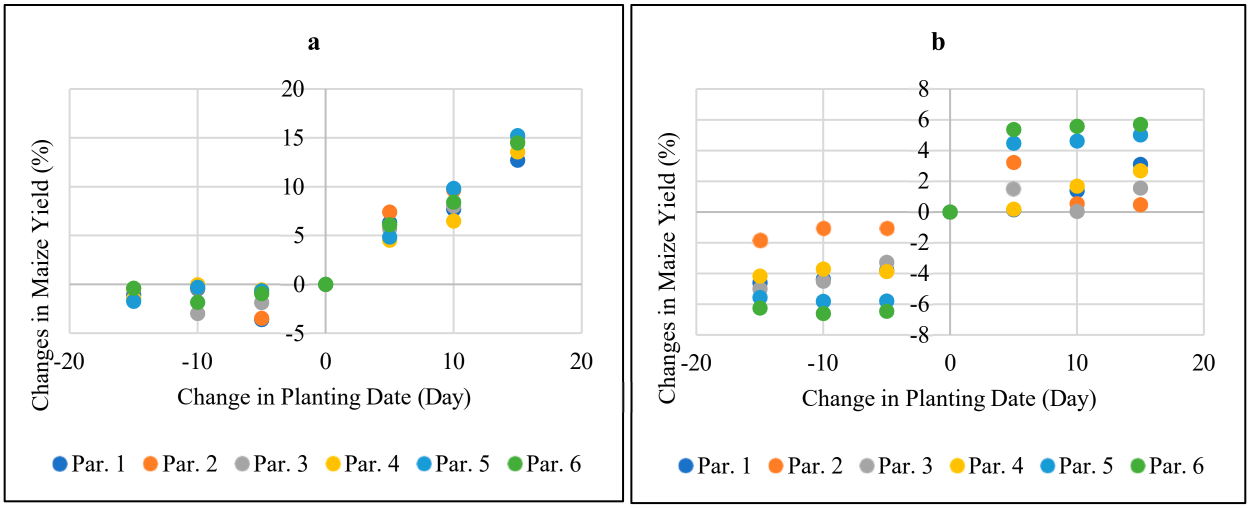

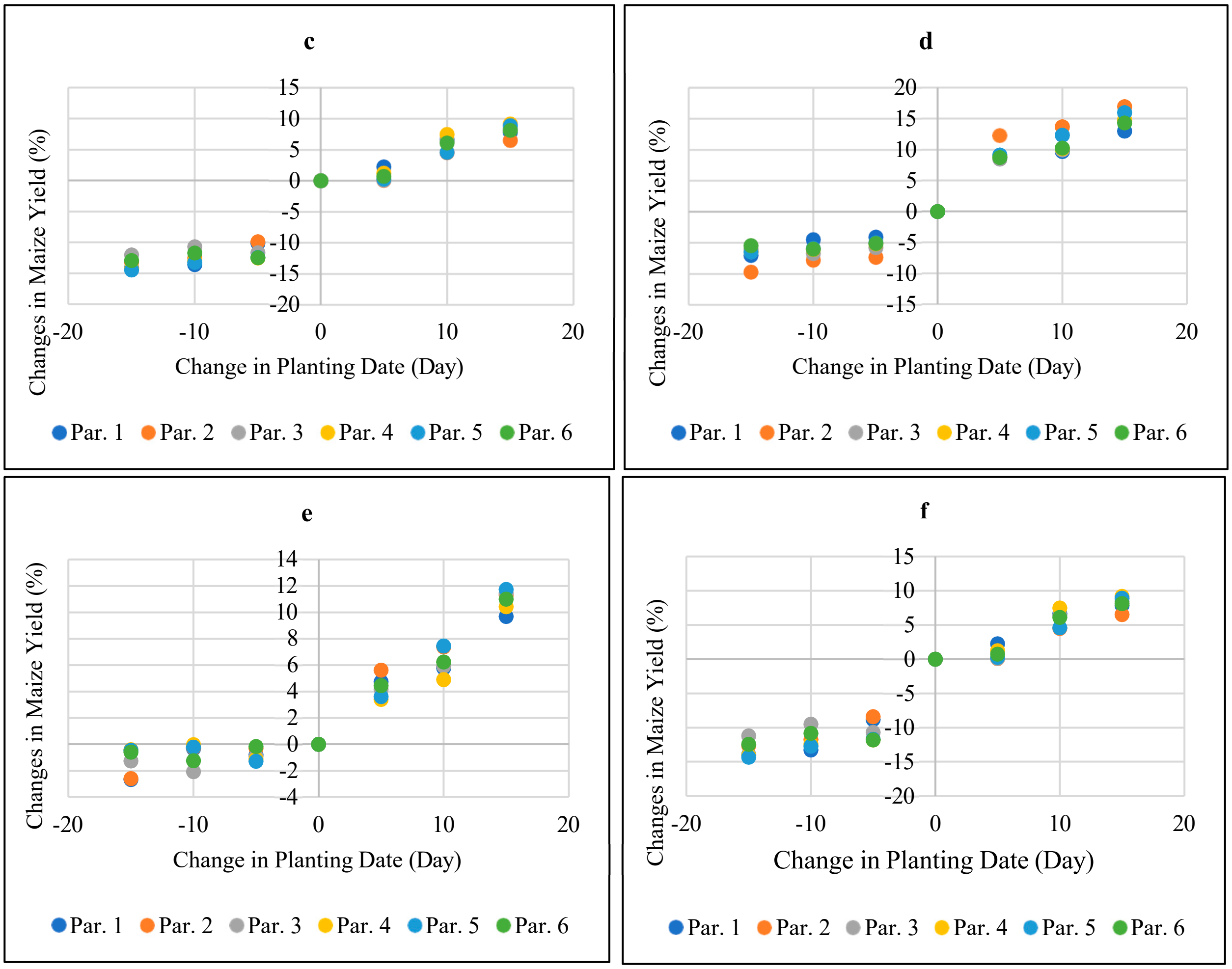

Fig. S21. Maize yield on the changed planting dates under RCP4.5 (2030s) under irrigation compared to the future maize yield without adaptation measures.

(Note: Station a~f denotes Hami, Changji, Hetian, Jiuquan, Jingyuan and Yulin, respectively)

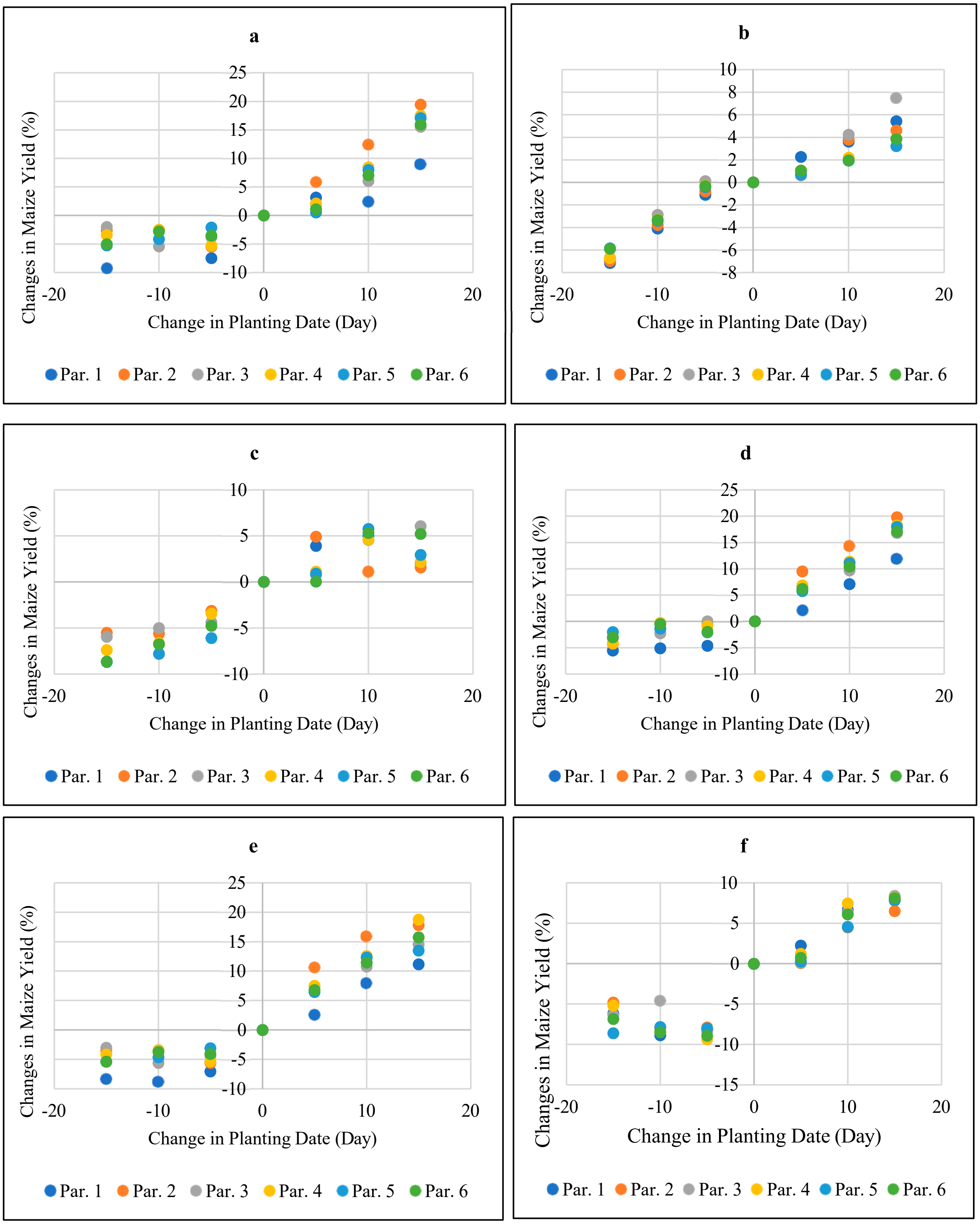

Fig. S22. Maize yield on the changed planting dates under RCP4.5 (2050s) under irrigation compared to the future

maize yield without adaptation measures.

(Note: Station a~f denotes Hami, Changji, Hetian, Jiuquan, Jingyuan and Yulin, respectively)

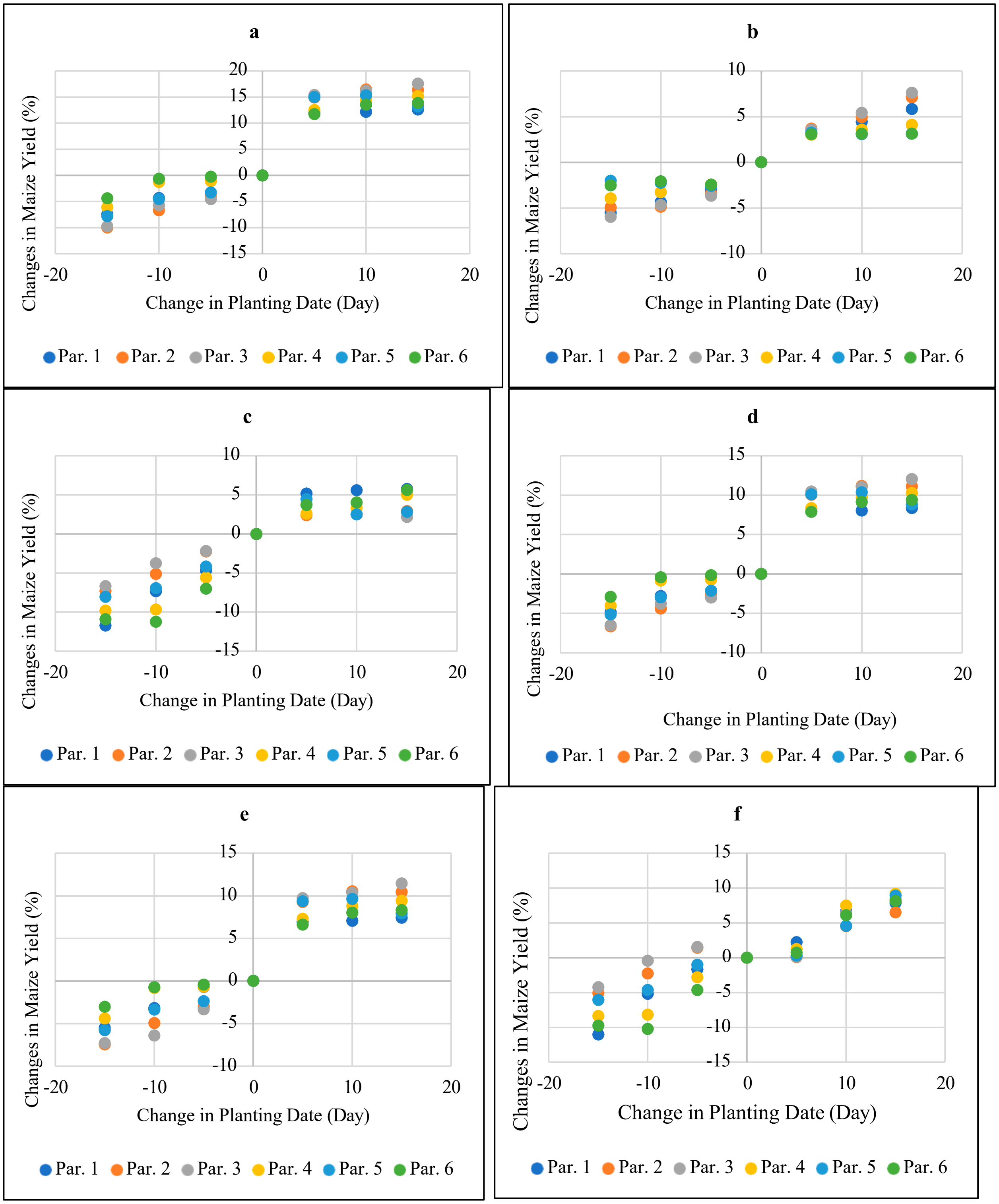

Fig. S23. Maize yield on the changed planting dates under RCP8.5 (2030s) under irrigation compared to the future maize yield without adaptation measures.

(Note: Station a~f denotes Hami, Changji, Hetian, Jiuquan, Jingyuan and Yulin, respectively)

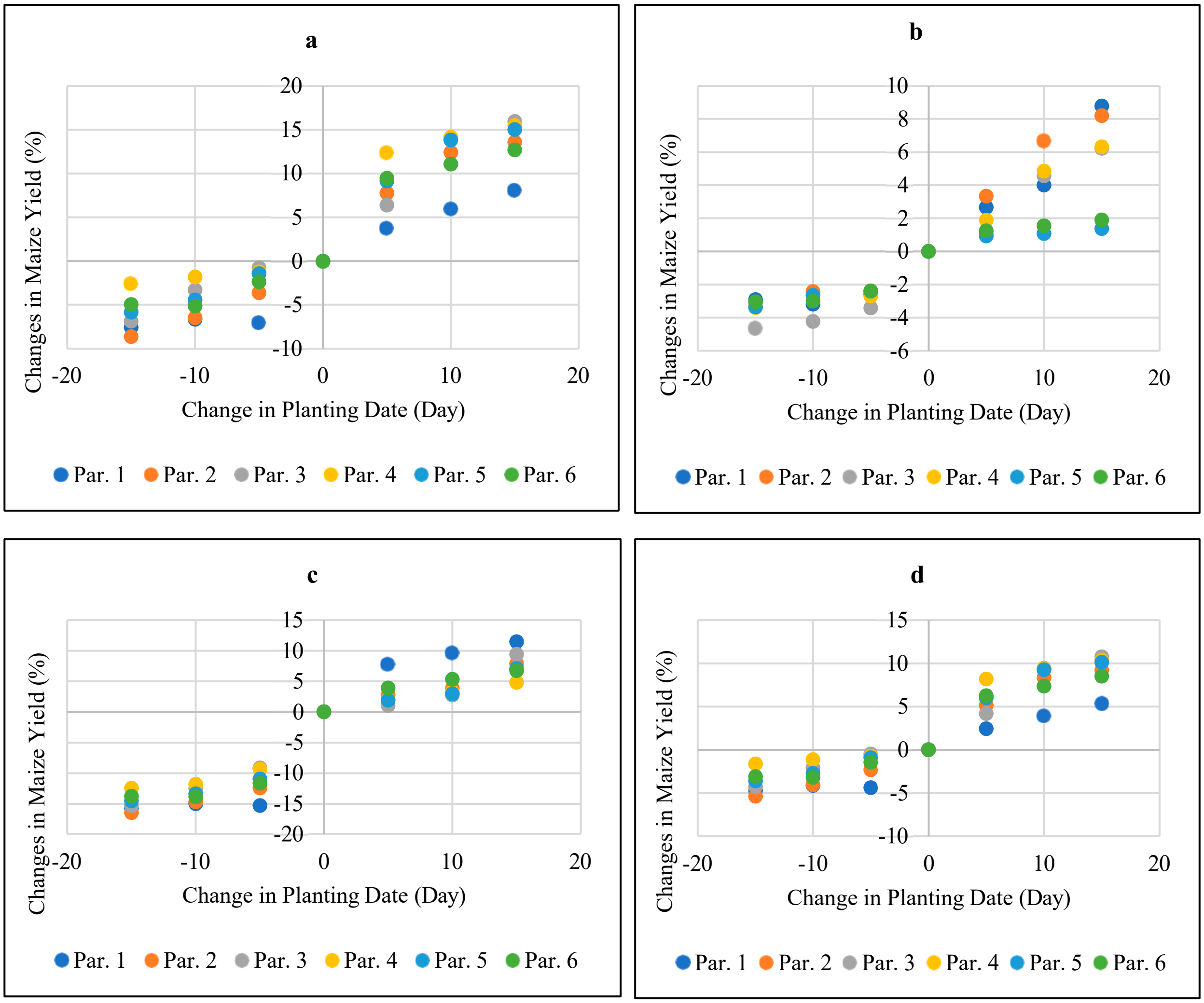

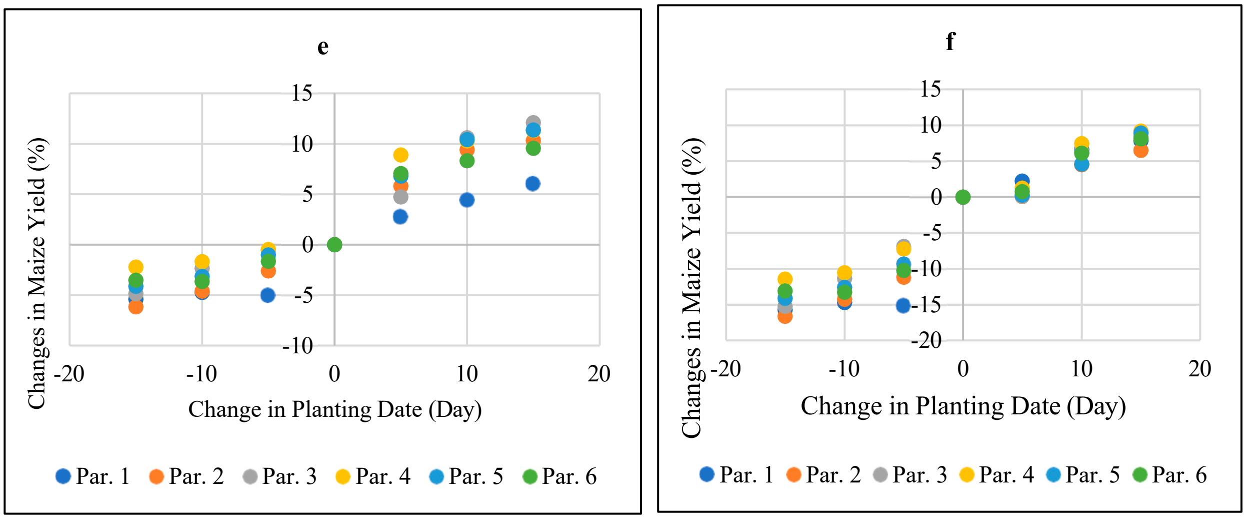

Fig. S24. Maize yield on the changed planting dates under RCP8.5 (2050s) under irrigation compared to the future maize yield without adaptation measures.

(Note: Station a~f denotes Hami, Changji, Hetian, Jiuquan, Jingyuan and Yulin, respectively)

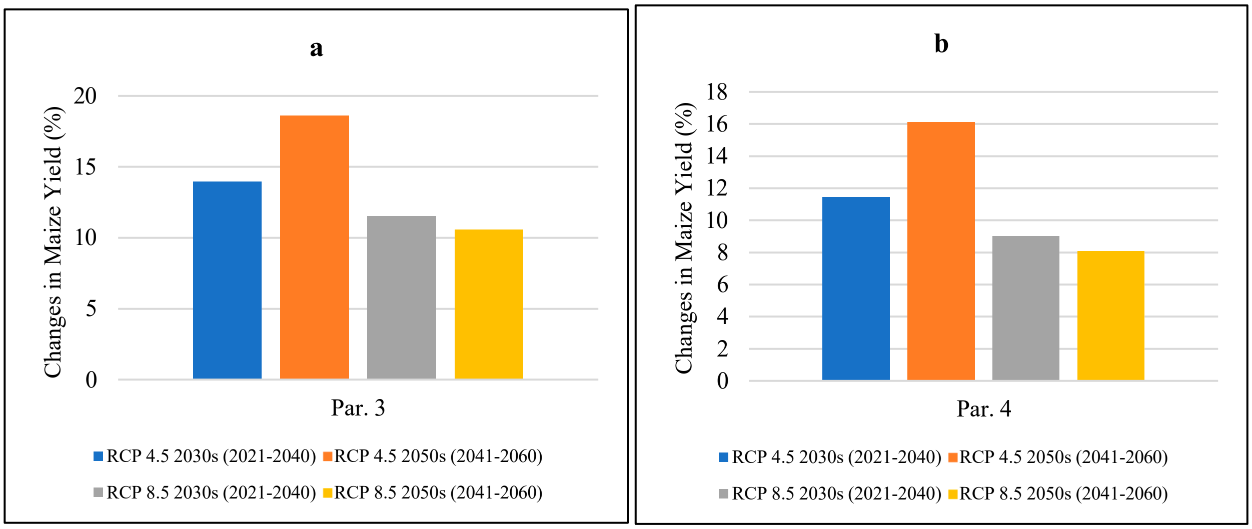

Fig. S25. Changes in maize yield (%) after cultivar change with Xinyu No.18 under future climate scenarios based on one set of optimal treatment parameter compared with the future maize yield without adaptation measures for a-Hami and b-Jingyuan.
